# Evolutionary game and simulation analysis of construction waste recycling from the perspective of stakeholders

**DOI:** 10.1371/journal.pone.0307652

**Published:** 2024-08-27

**Authors:** Yingchen Wang, Yan Liu, Tao Wang, Xiumin Xing, Xiaoxiao Geng

**Affiliations:** 1 School of Management Engineering and Business, Hebei University of Engineering, Handan, Hebei Province, China; 2 Handan First Hospital, Handan, Hebei Province, China; 3 School of Architecture and Art, Hebei University of Engineering, Handan, Hebei Province, China; University of the West of England, UNITED KINGDOM OF GREAT BRITAIN AND NORTHERN IRELAND

## Abstract

In current construction waste resource management processes, the effect of government supervision is unclear, and illegal treatment and low-quality reproduction of recycling and reprocessing enterprises by construction units are common. To improve the degree of resource utilization of construction waste and deeply explore the role of its key influencing factors, a tripartite evolutionary game model of construction waste resource treatment in which the government, construction units, and recycling and reprocessing enterprises are the research objects that considers public participation factors to be established. MATLAB is used to simulate the sensitivity of relevant parameters. The results show that: (1) An increase in government fines can regulate the behavior of enterprises; (2) Low government subsidies are conducive to the development of a tripartite stability strategy; and (3) An increase in the cost difference between the two strategies of the enterprise will weaken its willingness to carry out green operations (and after the cost difference exceeds the threshold, the enterprise will refuse to carry out green operations); (4) The reputation value brought by the public and the additional value added by reputation under the contrast effect have an incentive effect on the enterprise and the government; and (5) The peak value of the inverted U-shaped curve of government strategy choice is affected by the degree of public participation. Therefore, the government should propose rectifications in terms of fines and subsidies, and both companies can use technological innovation to reduce costs. At the same time, it is necessary to raise stakeholders’ awareness of resource utilization and encourage the public to actively participate in supervision. The research conclusions can provide a decision-making reference for improving the utilization of construction waste resources and the efficient treatment of construction waste resources.

## 1. Introduction

Construction waste is a key global issue [1]. Rapid urbanization has made it difficult to properly treat the large quantities of construction waste generated every year [1–3]. These wastes consist of a mixture of residual materials produced during construction, renovation, and demolition [4]. Worldwide, construction waste accounts for 30%-40% of the total urban solid waste [5]. However, compared with Japan, South Korea, Germany, and other countries whose resource utilization rate has reached more than 90%, in China, the resource utilization efficiency of solid waste in the construction industry is low [6]. Although the state has issued relevant policies to regulate the process of governance, there are still many types of solid construction waste that are landfilled or burned at will [7]. Construction waste management can improve environmental sustainability [8] and achieve a circular economy [1, 3, 9]. Therefore, how to address this resource scientifically and efficiently is necessary for the sustainable development of the construction industry.

Construction waste resource management has potential benefits and importance, but practical application presents multiple challenges. Inadequate and inefficient government supervision and policy guidance on construction and demolition waste management, which hinders the implementation of construction waste recycling, is one such challenge [10]. Furthermore, implementing construction waste recycling requires high costs for enterprises [11]. Meanwhile, conflicts of interest among stakeholders seriously affect the process of waste recycling [12] as parties tend to ignore the importance of the overall efficiency and long-term development of companies’ green operations [13]. Furthermore, driven by short-term profits, many enterprises have a negative attitude toward the recycling process of construction waste [14].

Construction waste management involves multiple stakeholders, who may have conflicting objectives that impact the construction waste recycling [1]. Previous studies have shown that the impact of stakeholders on China’s sustainable construction and waste management strategy is highly significant [15]. First, if the construction waste recycling process was controlled entirely by the government, then the costs would be high, while the effectiveness would be limited [3]. As information is disseminated across various levels, it may become distorted [16]. Public participation in management can improve this situation [18]. Second, the government is an important stakeholder in the recycling of construction waste [17]. On the one hand, the government can subsidize enterprises [19]; on the other hand, it can punish behaviors that are not conducive to the efficient treatment of construction waste. The state has launched the "Urban Construction Waste Management Regulations" to state that construction units should clear and transport the waste generated during construction in a timely manner to prevent environmental pollution [19]. Third, the construction unit is the source of construction waste, and the behavior of the construction unit seriously affects the efficiency of construction waste recycling to a certain extent. Finally, since the government encourages construction units to prioritize the use of construction waste recycling products, the issue of high-quality recycling and reprocessing enterprises is critical [15]. If the game relationship between the collective action of the government, construction units, and recycling and reprocessing enterprises in the resource utilization of construction waste from “nongreen” to “green operations” cannot be solved (here, “green operations” refer to the general designation of relevant operations such as the legal treatment of construction units and high-quality output from recycling and reprocessing enterprises), then the collaborative treatment system will not be able to efficiently use resources and implement recycling policies.

Most research in the field of construction waste recycling has focused on two aspects: construction waste treatment-related enterprises and the government [15, 20, 21]. Some studies have also explored the relationship between the public and waste disposal companies [16, 18, 22]. However, the literature has not considered the combined effects of the government, waste treatment enterprises (including construction units and recycling and reprocessing enterprises), and public participation. In addition, when discussing the resource utilization of construction waste, current research focuses more on whether recycling enterprises participate in this process [2, 23] but pays little attention to issues of quality in the recycling and reprocessing production process.

Therefore, this study aims to fill this academic gap. Our goal is to build a more comprehensive and in-depth analysis framework that can not only highlight the key role of the government in the process of construction waste recycling but also explore the role of waste treatment enterprises (i.e., construction units and recycling and reprocessing enterprises) and emphasize the importance of public participation. In this study, through in-depth analysis of the collaborative processing relationships among the government, construction units, and recycling and reprocessing enterprises, a three-party evolutionary game model that specifically considers the factor of public participation was constructed. This model not only discusses the strategic choice of production quality in the production process of recycling and reprocessing enterprises but also analyzes the impact of the public on the reputation of green operating enterprises under the “contrast effect.” In this study, a variety of factors, including public participation, are taken as important factors that affect the decision-making behavior of stakeholders in the construction waste recycling process, and are analyzed in detail. MATLAB software is used to simulate the evolution path of each subject’s strategy, and corresponding countermeasures and suggestions are proposed based on the simulation results to provide a more scientific and reasonable strategic reference for the utilization of construction waste resources.

## 2. Literature review

After reviewing the literature in this field, studies can be roughly categorized into three groups: research on the sustainable benefits of construction waste recycling, research on key stakeholders in the construction waste recycling industry, and research on evolutionary game theory.

### 2.1 Study on the resource utilization of construction waste

Construction waste refers to the waste bricks, concrete blocks, and other waste generated in new construction, reconstruction, expansion, and demolition of various buildings, structures, pipe networks, and decoration houses [24, 25]. Lilesh Gautam proposed using these industrial wastes as substitutes for concrete components [26]. Among them, 15% can be disposed of and turned into recycled building raw materials and 80% of the excavation earthwork can be used for the backfilling process and road laying in construction engineering [27]. Most construction waste treatment emphasizes three dimensions: reduction, recycling, and harmlessness [28].

The recycled blocks produced after crushing waste concrete, bricks, and tiles can meet the requirements of municipal road pavement and enclosure. Waste concrete is used in different grades of reinforced concrete structures after heating, mechanical friction, grinding, crushing, and screening [29, 30]. Lilesh Gautam proposed that bone china ceramic waste (BCCW) can be added to self-compacting concrete (SCC) as a partial substitute for cement [31]. The feasibility of BCPW and GW in the production of SCC was discussed [32], and the freshness, mechanical properties, and durability of SCC mixtures using these substances as substitutes for cement and fine aggregates were evaluated [33]. Some scholars have also focused on the influence of silicon powder on the workability and flexural tensile strength of concrete [34]. To improve construction waste recycling, the whole process of construction waste treatment can be considered in several stages: construction waste generation, disposal, resource product production, and sales [35]. After preliminary recycling, the construction waste is transported to the recycling center, and undergoes a separation system for separation, classification, recycling, and resource utilization. Feng Yahong clearly proposed that the goal of a deep recycling process, which can increase the recycling rate of construction waste to more than 60% [36]. Through field research and data analysis, Meng found that construction waste treatment faces the problems of outdated technology and the low added value of recycled products [37].

### 2.2 Key stakeholders and collaborative research

According to the research of Freeman and Li Yuanyuan, stakeholders involved in construction waste refer to the relevant personnel, departments, and enterprises that can affect this process and the final results [38, 39]. Many stakeholders participate in the recycling construction waste process [40]. After construction waste is generated, it is transported to recycling and reprocessing enterprises for reproduction, where new recycled products are formed, and then the waste is sold to construction units. This process includes a recycling and remanufacturing system composed of construction units and recycling and reprocessing enterprises as the main stakeholders [41]. Based on the case of construction waste disposal in Shanghai, Lin Chun found that there are many subjects involved in construction waste disposal systems and proposed an architecture of construction waste disposal management and operation [42]. With the upgrading of the processing model, domestic and foreign scholars have moved beyond the government-led model and proposed the concept of multi-subject collaborative processing [43]. Using a simulation in Shanghai as an example, Su et al. constructed an evolutionary game model of decision-making behavior among local governments, contractors, and recycling factories in the process of construction waste recycling to understand the behavior, needs, and synergies of corporate stakeholders [15].

Construction units are the main producers of construction waste. Shao Zhiguo analyzed the interests of the construction unit in detail [43, 44]. In analyzing the current situation of construction waste recycling China and abroad, Li Cong and Zhang Xin clearly noted the importance of construction units in the process of construction waste treatment [45].

Recycling and reprocessing enterprises, also known as resource recycling enterprises, are the key stakeholders in the process of construction waste recycling. Jiang Mingyang described in detail the importance of resource recycling enterprises for waste resource treatment by constructing the whole life cycle resource industry chain of construction waste [46]. Long Hongyu mentioned in a study that the production of recycled products by recycling units is a key link in the recycling process of construction demolition waste, and the quality of the produced products is affected by the government’s reward and punishment mechanism to a certain extent [47]. Yangyue Su used the Shanghai recycled brick market as an example to determine the factors that influence the strategic choice of recycling and reproduction enterprises [15].

The government is responsible for supervision and policy making. Stakeholder engagement and key strategies are highly relevant to waste management for demolition, with the government being the most important stakeholder [1]. Ma and Zhang mentioned that government subsidies to construction companies are crucial for promoting the recycling of construction waste in China [48]. From the perspective of government rewards and punishments, social capital under various subsidized financing systems present different strategies for waste disposal [49]. Tan Ruwen focused on the impact of government subsidies on the technological innovation of resource-based enterprises [50]. Lu Mei and Huang Zhong constructed a two-party game model in which the government was a participant, re-emphasizing the important position of the government in the process of recycling construction waste [51]. Long Hongyu proposed that the government’s fines can effectively control the amount of construction waste generated and control the quality of recycled products [47].

The public is the supervisor of the construction waste recycling process. Based on the perspective of the public, Yang Su and Yao Lichun constructed an analysis model of public participation in the resource utilization of construction waste and explained how the public can effectively serve a supervisory role in the recycling process of construction waste [16]. Yao Lichun proposed that appropriate public participation can compensate for the shortcomings and defects of the government in decision-making related to construction waste resource management [52].

### 2.3 Application of evolutionary game theory in construction waste treatment

Compared with traditional games, evolutionary game theory has the characteristics of bounded rationality and dynamic balance, which are closer to reality and more suitable for complex situations in reality [53–56]. Li Songliang noted that the evolutionary game method is more in line with the repeated game relationship between collaborative innovation subjects with both cooperation and conflict [57]. Yuan Hongping and Wang Zhuoping constructed a game model with or without government subsidies, reflecting that government intervention has an impact on evolutionary stability [21]. Lu Mei and Huang Zhong constructed an evolutionary game between the government and construction waste emission units and between the government and construction waste recycling units [51]. Xue Fei applied system dynamics to construct a construction waste recycling model with PPP projects, reflecting the impact of various factors on the optimal ESS [58]. Xia Haofan described the relationship between engineering construction units and waste treatment enterprises, established a game model, and analyzed the sensitivity of related factors [59].

### 2.4 Literature review

While many researchers have begun to carry out more detailed research on the development of the construction waste recycling industry, these studies have certain limitations. The construction waste recycling process encompasses the entire process of generation, treatment, recycling, and reprocessing. With growing awareness of environmental protection, public participation in social governance has had a positive effect [16, 60].

Most research has focused on the relationships between construction waste treatment-related enterprises and the government [15, 20, 21] or between the public and waste treatment-related enterprises [16, 18, 22]. Few studies have taken into account the importance of government participation and involved waste-related treatment enterprises, namely, construction units and recycling and reprocessing enterprises, and considered the importance of public participation factors [2, 23]. In addition, most of the current research in this area focuses on analyzing whether recycling and reprocessing enterprises participate in the resource utilization process of construction waste. Few articles have focused on the production quality of recycling and reprocessing enterprises. According to the literature review, the high-quality reproduction of recycling and reprocessing enterprises plays a very important role in the recycling process of construction waste.

Therefore, this paper analyses the utilization of construction waste resources under the consideration of public participation from the perspective of improving the utilization of construction waste resources. Therefore, this paper constructs a tripartite evolutionary game model composed of the government, construction units, and recycling and reprocessing enterprises that considers their collaborative processing relationships under public participation. The selection of production quality strategy in the reproduction process of recycling and reprocessing enterprises is studied. The influence of public reputation on green operation under the “contrast effect, ” where “green operation” generally refers to relevant operations such as the legal treatment of the construction unit and high-quality production of the recycling and reprocessing enterprise) is taken as one of the parameters of this paper. The influence of many factors, including public participation, on the decision-making behavior of stakeholders in the process of construction waste recycling is analyzed. On this basis, this paper simulates and analyzes the evolution path of each subject strategy through MATLAB and proposes corresponding countermeasures and suggestions according to the simulation results.

## 3. Model construction

The process of resource utilization of construction waste requires the collaboration of many subjects. Using the government, construction units, and recycling and reprocessing enterprises as the research subjects, this paper constructs an evolutionary game model to explore the behavior strategies of the above three parties under the factor of public participation.

### 3.1 Evolutionary game theory

Evolutionary game theory combines the knowledge of biological evolution theory with traditional game theory. Considering real world conditions, the decision-making of each participant is put forward under the premise of bounded rationality. Each participant can dynamically adjust its own strategy according to changes in the internal and external environment. Through the mutual influence and interaction mechanism between participants, the evolution results approach real conditions through continuous learning and adjustment. The purpose of evolutionary game theory is to find the optimal evolutionary stable strategy (ESS) for the system group. The basic idea of the ESS is as follows: if the payoff of the small group in the mixed group is greater than the payoff of the individual in the original group, then the small group will have the ability to invade the large group; in contrast, they cannot invade large groups and tend to disappear in the process of evolution.

This paper reviews the collaborative relationships among construction waste stakeholders, screens the influencing factors, and constructs an evolutionary game model of construction waste resource treatment. Then, the ESS is solved by means of the expectation function, replicator dynamic equation, and Jacobian matrix. Finally, the influence of relevant parameters on the optimal ESS is analyzed.

### 3.2 Analysis of the collaborative mechanism of construction waste recycling

When the construction unit legally handles its waste and the recycling and reprocessing enterprise has high-quality output, the construction waste resource utilization system, including the construction unit and the recycling and reprocessing enterprise, can effectively and efficiently treat construction waste and reduce the problem of increased carbon emissions caused by the illegal disposal of waste. First, the construction unit sells the reusable construction waste to the recycling and reprocessing enterprises at a low price, and then it can purchase high-quality recycled products from the recycling and reprocessing enterprises, which is conducive to improving the quality of new construction products. The collaborative mechanism of construction waste recycling is shown in S1 Fig.

Government authorities are the key subjects of urban environmental governance, and they are also policy makers and supervisors in the process of environmental management. In the tripartite evolutionary game model of government-construction unit-recycling and reprocessing enterprises, government authorities play an important role as regulators and leaders in construction waste resource management. The government regulates the behavioral strategies of construction units and recycling and reprocessing enterprises. To increase compliance with construction waste treatment, the government provides certain subsidies and punishments according to behavior. As a part of governance, these policies can foster construction waste recycling so that construction waste can be efficiently utilized and benefits can be obtained. It also ensures the green and low-carbon development of the construction industry and yields environmental benefits. Strict government regulation promotes the efficient use of waste and improving the environment, which can increase the public’s sense of identity.

When a construction unit legally disposes waste, construction waste will not arbitrarily end up in landfills, which can reduce environmental pollution and yield environmental benefits [44]. However, at this time, with rising treatment costs on the part of the construction unit, recycling and reprocessing enterprises can reduce high recycling costs by obtaining legally processed waste from the construction unit. When construction waste is illegally disposed, the treatment cost will decrease, carbon emissions will increase, recycling and reprocessing enterprises cannot directly obtain construction waste from the construction unit, and the material purchase cost will increase. Low-quality recycled products are easily damaged during transportation and use. Therefore, its effective utilization rate is relatively low, and the components that cannot be reused result in a waste of resources [15]. In low-quality waste reproduction, because the technical equipment available is not sufficiently advanced, it is difficult to purify the residual waste during reprocessing, which can cause severe environmental pollution. High-quality output from recycling and reprocessing enterprises can improve the utilization rate of construction waste, reduce carbon emissions, and produce good-quality building materials. The government encourages construction units to use remanufactured building materials, so high-quality recycled products can improve the quality of buildings built by construction units. The government regulates the behavior of enterprises, and public participation affects the credibility of the government and the reputation of enterprises.

The public is not only the consumer and evaluator of social services but also the producer of services [61]. With the continuous advancement of deliberative democracy, the public has sufficient legitimate and reasonable justifications to participate in construction waste resource management. However, due to the limited information acquisition channels of government authorities, information is easily distorted in its transmission, which results in the untimely discovery of problems. At this time, public participation can solve this problem to a certain extent. Involving the public can help detect environmental destruction in a timely manner [62].

Therefore, an evolutionary game model of construction waste recycling composed of the government, construction units, and recycling and reprocessing enterprises is proposed, and the strategic choices of game players in the model considering public participation factors are explored. Through the above analysis, it can be seen that clarifying the interests of the various subjects and determining the relationships between various stakeholders can lay the foundation for collaborative construction waste treatment. The relationships between stakeholders are shown in S2 Fig.

### 3.3 Basic assumptions and parameter settings

To improve the recycling rate of construction waste, a collaborative treatment system should be implemented. The waste treatment methods used by construction units and recycling and reprocessing enterprises, as the main actors of recycling, directly affect China’s ecological environment. Therefore, government regulation is very important and can guide construction enterprises to legally address construction waste and recycling and reprocessing enterprises to carry out high-quality recycling, which can improve the resource utilization efficiency of construction waste. However, as an important supplement to the management and control of illegal treatment and low-quality recycling, the public can effectively improve the regulatory efficiency of government authorities.

According to the characteristics of the stakeholders involved in construction waste recycling, the following assumptions and parameters are proposed. The specific meaning of the parameters is shown in Table 1:

**Table 1:** Parameter table.

stakeholders	Parameter	The meaning of the expression of the selected parameters
**Government**	*ΔC*_1_	The increase in the cost of government regulation than nonregulation
	*S*_*1*_	Subsidies given to construction units during government regulation
	*S*_2_	Subsidies for recycling and reprocessing enterprises in government regulation
	*F*_1_	The government has fined construction units for illegal disposal of construction waste.
	*F*_2_	The government gives recycling and reprocessing enterprises a fine for low-quality production.
	*P*_1_	The benefits of government regulation in terms of credibility when the public participates in supervision
	*R*_1_	The environmental benefits increased by the legal treatment of the construction unit compared with the illegal treatment
	*R*_2_	The environmental benefits of high-quality reproduction in recycling and reprocessing enterprises are higher than those of low-quality reproduction.
	*R*	The environmental benefits brought by the green operation of both parties (the construction unit legally treatment and the high-quality reproduction of the recycling and reprocessing enterprise) are better than those brought by the non-green operation of both parties. (R1+R2≠R)
**construction unit**	*ΔC*_2_	The cost increase of legal treatment of construction unit is greater than that of illegal treatment.
	*P*_2_	When the construction unit is legally handled, public participation in supervision makes the company gain in terms of reputation and stocks.
	Δ*P*	Only when the construction unit has green operations, public participation in supervision enables the construction unit to gain additional value added in terms of reputation, reputation, and stock.
	*α*	The ratio of high-quality materials purchased from recycling and reprocessing enterprises to the improvement of building quality compared with ordinary materials
	*M*_1_	Degree of building quality
**Recycling and reprocessing enterprises**	*ΔC*_3_	The cost increase of high-quality reproduction of recycling and reprocessing enterprises is higher than that of low-quality reproduction.
	*P*_3_	In the high-quality reproduction of recycling and reprocessing enterprises, public participation in supervision enables enterprises to obtain benefits in terms of reputation and stocks.
	Δ*P*	Only when recycling and reprocessing enterprises engage in green operations does public participation in supervision makes them gain additional value added, such as in terms of reputation and stock.
	*β*	The ratio of the price of raw materials purchased by the recycling and reprocessing enterprises directly from the construction unit to the price of raw materials purchased by other channels.
	*M*_2_	Recycling and reprocessing enterprises purchase the cost of recyclable construction waste from other channels.

Hypothesis 1: The construction waste studied in this paper refers to waste that cannot be directly recovered and can be processed by complex treatment processes.
Hypothesis 2: This paper assumes that the participants have bounded rationality.
Hypothesis 3: This paper assumes that the transformation process of each subject’s strategy is gradual, so it is represented by the replication dynamic equation formula.
Hypothesis 4: The choice of each subject’s strategy depends on the benefits under each strategy.
Hypothesis 5: The government has two strategic choices, regulation and regulation, with very low probability of nonregulation. The probability of regulation is *x*(0≤*x*≤1), and the probability of nonregulation is 1−*x*.
Hypothesis 6: There are two kinds of construction unit strategies: legal treatment and illegal treatment. The probability of legal treatment is *y*(0≤*y*≤1), and the probability of illegal treatment is 1-*y* The parameter *β* is the ratio of the price of raw materials purchased by the recycling and reprocessing enterprises directly from the construction unit to the price of raw materials purchased by other channels (0<*β*<1).
Hypothesis 7: The strategies of recycling and reprocessing enterprises include high-quality reproduction and low-quality reproduction. The probability of high-quality reproduction is *z*(0≤*z*≤1), and the probability of low-quality reproduction is 1−*z*. The parameter *α* is the ratio of high-quality materials purchased from recycling and reprocessing enterprises to the improvement in building quality compared with that of ordinary materials.
Hypothesis 8: Assuming that the government does not regulate but that the public participates in supervision, when only one of the two parties still adheres to the original intention for green operation [60], there will be additional value added in terms of reputation, prestige, and stock due to the contrast effect, and the parameter is expressed as Δ*P*

### 3.4 Construction of the evolutionary game model

According to the above parameter settings and the relationships among the game subjects, a tripartite evolutionary game model matrix including the government, construction units, and recycling and reprocessing enterprises is constructed, as shown in Table 2.

**Table 2:** Payoff matrix.

			Recycling and reprocessing enterprises	
			High-quality reproduction (*Z*)	low-quality reproduction (1−*z*)
**The Government, the construction unit**	Regulation (*x*)	Legal treatment (*y*)	*R*+*P*_1_-*S*_1_-*S*_2_-Δ*C*_1_ *S*_1_+*αM*_*1*_-Δ*C*_2_*+P*_2_ *S*_2_+(1−*β*)*M*_2_-Δ*C*_3_*+P*_3_	*R*_*1*_+*P*_1_+*F*_2_-Δ*C*_*1*_-*S*_1_-*S*_2_- *S*_1_+*P*_2_-Δ*C*_*2*_ *S*_2_+(1−*β*)*M*_2_-*F*_3_
		Illegal treatment (1−*y*)	*R*_2_+*F*_1_+*P*_1_-*S*_1_-*S*_2_-Δ*C*_1_ *S*_1_+*αM*_1_−*F*_1_ *S*_2_+*P*_3_−Δ*C*_3_	*P*_1_+*F*_1_+*F*_2_-*S*_1_-*S*_2_-Δ*C*_1_ *S*_1_−*F*_1_ *S*_2_−*F*_2_
	Nonregulation (1-*x*)	Legal treatment (*y*)	*R* *αM*_1_+*P*_2_−Δ*C*_2_ (1−*β*)*M*_2_+*P*_3_-Δ*C*_3_	*R*_1_ *P*_2_+Δ*P*−Δ*C*_2_ (1−*β*)*M*_2_
		Illegal treatment (1−*y*)	*R*_2_ *αM*_1_ *P*_3_+Δ*P*−Δ*C*_3_	0 0 0

## 4. Model analysis

### 4.1 Expected revenue function of game players

The expected benefits of the government’s choice of regulation and nonregulation are as follows:

$$\begin{array}{l} E_{11}=yz\left[R-S_{1}-S_{2}-\Delta C_{1}+P_{1}\right]\\ +y(1-z)\left[R_{1}-S_{1}-S_{2}-\Delta C_{1}+F_{2}+P_{1}\right]\\ +(1-y)z\left[R_{2}-S_{1}-S_{2}-\Delta C_{1}+F_{1}+P_{1}\right]\\ +(1-y)(1-z)\left[F_{1}+F_{2}-S_{1}-S_{2}-\Delta C_{1}+P_{1}\right] \end{array}$$ (1)

$$E_{12}=yz[R]+y(1-z)[R_{1}]+(1-y)z[R_{2}]$$ (2)

The average expected return of government strategy choice is:

$$\bar{E_{1}}=xE_{11}+(1-x)E_{12}$$ (3)

The expected benefits of the construction unit’s choice of legal treatment and illegal treatment are as follows:

$$E_{21}=xz\Delta P+x(S_{1}-\Delta P)+z[(1-\alpha )M_{1}-\Delta P]-\Delta C_{2}+P_{2}+\Delta P$$ (4)

$$E_{22}=x(S_{1}-F_{1})+z(1-\alpha )M_{1}$$ (5)

The average expected return of the construction unit’s strategy selection:

$$\bar{E_{2}}=yE_{21}+(1-y)E_{22}$$ (6)

The expected benefits of high-quality reproduction and low-quality reproduction in recycling and reprocessing enterprises are as follows:

$$E_{31}=xy\Delta P+x(S_{2}-\Delta P)+y[(1-\beta )M_{2}-\Delta P]-\Delta C_{3}+P_{3}+\Delta P$$ (7)

$$E_{32}=x(S_{2}-F_{2})+y(1-\beta )M_{2}$$ (8)

The average expected return of recycling and reprocessing enterprises is:

$$\bar{E_{3}}=zE_{31}+(1-z)E_{32}$$ (9)

### 4.2 Stability analysis

The replication dynamic equation of the government regulation strategy is:

$$\begin{array}{l} F(x)=dx/dt=x(E_{11}-\bar{E_{1}})=x(1-x)(E_{11}-E_{12})\\ =x(1-x)(-yF_{1}-zF_{2}+F_{1}+F_{2}-S_{1}-S_{2}-\Delta C_{1}+P_{1}) \end{array}$$ (10)

Let *G*(*y*) = *yF*_1_−*zF*_2_+*F*_1_+*F*_2_−*S*_1_−*S*_2_−Δ*C*_1_+*P*_1_. To ensure that the probability that the government department will choose the regulatory strategy is stable, *F*(*x*) = 0 and $\frac{dF\left(x\right)}{dx}<0$ must be satisfied.

Let *F*(*x*) = *dx*/*dt* = 0, the obtained solution may be the equilibrium point of the evolution process.

If $y=y^{*}=\frac{-zF_{2}+F_{1}+F_{2}-S_{1}-S_{2}-\Delta C_{1}+P_{1}}{F_{1}}$, *F*(*x*)≡0, at this time, then regardless of the value of *x*, *F*(*x*) constant is zero, the government’s strategy does not change with time.

If *y*<y*, *G*(*y*)>0, $\frac{dF\left(x\right)}{dx}{|}_{x=0}<0$, *x* = 0 is the stable equilibrium solution of *F*(*x*); i.e., then the government will tend to adopt a nonregulatory strategy.

If *y*<y*, $\frac{dF\left(x\right)}{dx}{|}_{x=1}<0$, *x* = 1 is the stable equilibrium solution of *F*(*x*); i.e., the government tends to follow the regulation strategy.

The replication dynamic equation of the construction unit’s legal treatment strategy is:

$$\begin{array}{l} F(y)=dy/dt=y(E_{21}-\bar{E_{2}})=y(1-y)(E_{21}-E_{22})\\ =y(1-y)(xz\Delta P+x\left(-\Delta P+F_{1}\right)-z\Delta P-\Delta C_{2}+P_{2}+\Delta P) \end{array}$$ (11)

Let *G*(*z*) = *xz*Δ*P*+*x*(−Δ*P*+*F*_1_)−*z*Δ*P*−Δ*C*_2_+*P*_2_+Δ*P*; in the same way, the following can be obtained:

If $z=z^{*}=\frac{x\left(-\Delta P+F_{1}\right)-\Delta C_{2}+P_{2}+\Delta P}{\Delta P-x\Delta P}$ and *F*(*y*)≡0, regardless of whether *y* or *F*(*y*) constant is zero, the construction unit strategy does not change with time.

If *z*<z*, *G*(*z*)>0, $\frac{dF\left(y\right)}{dy}{|}_{y=0}<0$, then *y* = 0 is the stable equilibrium solution of *F*(*y*).

If *z*>z*, $\frac{dF\left(y\right)}{dy}{|}_{y=1}<0$, then *y* = 1 is the stable equilibrium solution of *F*(*y*).

The replication dynamic equation of the high-quality reproduction strategy of recycling and reprocessing enterprises is:

$$\begin{array}{l} F(z)=dz/dt=z(E_{31}-\bar{E_{3}})=z(1-z)(E_{31}-E_{32})\\ =z(1-z)(xy\Delta P+x\left(-\Delta P+F_{2}\right)-y\Delta P-\Delta C_{3}+P_{3}+\Delta P) \end{array}$$ (12)

Let *G*(*x*) = *xy*Δ*P*+*x*(−Δ*P*+*F*_2_)−*y*Δ*P*−Δ*C*_3_+*P*_3_+Δ*P*; in the same way, the following can be obtained:

If $x=x^{*}=\frac{y\Delta P+\Delta C_{3}-P_{2}-\Delta P}{y\Delta P-\Delta P+F_{2}}$ and *F*(*z*)≡0, at this time, regardless of the value of z, *F*(*z*) is constant at zero.

If *x*<x*, *G*(*x*)>0, $\frac{dF\left(z\right)}{dz}{|}_{z=0}<0$, then *z* = 0 is the stable equilibrium solution of *F*(*z*).

If *x*>x*, $\frac{dF\left(z\right)}{dz}{|}_{z=1}<0$, then *z* = 1 is the stable equilibrium solution of *F*(*z*).

### 4.3 Equilibrium strategy analysis of the evolutionary game model

From *F*_*x*_(*x*, *y*, *z*) = 0, *F*_*y*_(*x*, *y*, *z*) = 0, and *F*_*z*_(*x*, *y*, *z*) = 0, we can obtain eight pure strategy equilibrium points of the evolution system: *E*_1_(0, 0, 0), *E*_2_(0, 1, 0), *E*_3_(0, 0, 1), *E*_4_(1, 0, 0), *E*_5_(1, 0, 1), *E*_6_(1, 1, 0), *E*_7_(0, 1, 1), *E*_8_(1, 1, 1). According to Friedman’s method, the stability of the equilibrium point of the differential system can be obtained via eigenvalue analysis of the Jacobian matrix. The Jacobi matrix of the tripartite evolutionary game is as follows:

$$\begin{array}{c} J=\left[\begin{array}{ccc} \frac{\partial F\left(x\right)}{\partial x} & \frac{\partial F\left(x\right)}{\partial y} & \frac{\partial F\left(x\right)}{\partial z}\\ \frac{\partial F\left(y\right)}{\partial x} & \frac{\partial F\left(y\right)}{\partial y} & \frac{\partial F\left(y\right)}{\partial z}\\ \frac{\partial F\left(z\right)}{\partial x} & \frac{\partial F\left(z\right)}{\partial y} & \frac{\partial F\left(z\right)}{\partial z} \end{array}\right]\\ =\left[\begin{array}{ccc} \left(1-2x\right)G\left(y\right) & x\left(x-1\right)F_{1} & x\left(x-1\right)F_{2}\\ y\left(1-y\right)\left(z\Delta P-\Delta P+F_{1}\right) & \left(1-2y\right)G\left(z\right) & y\left(1-y\right)\left(x\Delta P-\Delta P\right)\\ z\left(1-z\right)\left(y\Delta P-\Delta P+F_{2}\right) & z\left(1-z\right)\left(x\Delta P-\Delta P\right) & \left(1-2z\right)G\left(x\right) \end{array}\right] \end{array}$$ (13)

According to the hypothesis of the size of the model parameters, *P*_1_<*S*_1_+*S*_2_+Δ*C*_1_<*P*_1_+*F*_1_+*F*_2_, *S*_1_+*S*_2_+Δ*C*_1_<*P*_1_+*F*_1_+*F*_2_; therefore, *E*_1_(0, 0, 0), *E*_5_(1, 0, 1), and *E*_8_(1, 1, 1) are not stable points.

After analysis, the stability and judgment conditions of the eight equilibrium points are shown in Table 3. In addition to *E*_1_(0, 0, 0), *E*_5_(1, 0, 1), and *E*_8_(1, 1, 1), the other six equilibrium points can evolve into a stable state when the corresponding conditions are satisfied, as shown in Table 4. *E*_1_(0, 0, 0) and *E*_8_(1, 1, 1) are unstable points, while *E*_4_(1, 0, 0) and *E*_7_(0, 1, 1) are asymptotically stable points. This indicates that when the construction unit illegally disposes waste and the recycling and reprocessing enterprises produce low-quality output, the government will participate in regulation to avoid the construction waste recycling process falling into a vicious circle. However, when the construction unit legally treats its waste and recycles the high-quality output from the reprocessing enterprise, the government will tend to relax regulations to control the unnecessary cost of human and material resources in the regulation process. This reflects that the government cannot overregulate, and overregulation may also have adverse effects.

**Table 3:** Stability analysis of the pure strategy equilibrium point.

Evolutionary equilibrium point	*λ*_1_	*λ*_2_	*λ*_3_	Characteristic root symbol	Stability conclusion
*E*_1_(0, 0, 0)	*F*_1_+*F*_2_−*S*_1_−*S*_2_−Δ*C*_1_+*P*_1_	−Δ*C*_2_+*P*_2_+Δ*P*	−Δ*C*_3_+*P*_3_+Δ*P*	+, /, /	unstable point
*E*_2_(0, 1, 0)	*F*_2_−*S*_1_−*S*_2_−Δ*C*_1_+*P*_1_	Δ*C*_2_−*P*_2_−Δ*P*	−Δ*C*_3_+*P*_3_	/, /, /	asymptotic stable point
*E*_3_(0, 0, 1)	*F*_1_−*S*_1_−*S*_2_−Δ*C*_1_+*P*_1_	−Δ*C*_2_+*P*_2_	Δ*C*_3_−*P*_3_−Δ*P*	/, /, /	asymptotic stable point
*E*_4_(1, 0, 0)	−*F*_1_−*F*_2_+*S*_1_+*S*_2_+Δ*C*_1_−*P*_1_	*F*_1_−Δ*C*_2_+*P*_2_	*F*_2_−Δ*C*_3_+*P*_3_	-, /, /	asymptotic stable point
*E*_5_(1, 0, 1)	−*F*_1_+*S*_1_+*S*_2_+Δ*C*_1_−*P*_1_	*F*_1_−Δ*C*_2_+*P*_2_	*−F*_2_+Δ*C*_3_−*P*_3_	+, /, /	unstable point
*E*_6_(1, 1, 0)	−*F*_2_+*S*_1_+*S*_2_+Δ*C*_1_−*P*_1_	−*F*_1_+Δ*C*_2_−*P*_2_	*F*_2_−Δ*C*_3_+*P*_3_	/, /, /	asymptotic stable point
*E*_7_(0, 1, 1)	−*S*_1_−*S*_2_−Δ*C*_1_+*P*_1_	Δ*C*_2_−*P*_2_	Δ*C*_3_−*P*_3_	-, /, /	asymptotic stable point
*E*_8_(1, 1, 1)	*S*_1_+*S*_2_+Δ*C*_1_−*P*_1_	−*F*_1_+Δ*C*_2_−*P*_2_	−*F*_2_+Δ*C*_3_−*P*_3_	+, /, /	unstable point

**Table 4:** Comparison of asymptotically stable points.

Evolutionary equilibrium point	Government stability conditions	Stability conditions of construction units	Stable conditions of recycling and reprocessing enterprises
*E*_2_(0, 1, 0)	*P*_1_+*F*_2_<*S*_1_+*S*_2_+Δ*C*_1_	Δ*C*_2_<*P*_2_+Δ*P*	*P*_3_<Δ*C*_3_
*E*_3_(0, 0, 1)	*P*_1_+*F*_1_<*S*_1_+*S*_2_+Δ*C*_1_	*P*_2_<Δ*C*_2_	Δ*C*_3_<*P*_3_+Δ*P*
*E*_4_(1, 0, 0)	/	*P*_2_+*F*_1_<Δ*C*_2_	*P*_2_+*F*_2_<Δ*C*_3_
*E*_6_(1, 1, 0)	*S*_1_+*S*_2_+Δ*C*_1_<*P*_1_+*F*_2_	Δ*C*_2_<*P*_2_+*F*_1_	*P*_2_+*F*_2_<Δ*C*_3_
*E*_7_(0, 1, 1)	*P*_1_<*S*_1_+*S*_2_+Δ*C*_1_	Δ*C*_2_<*P*_2_	Δ*C*_3_<*P*_3_

Scenario 1: When the conditions satisfy *P*_1_+*F*_2_<*S*_1_+*S*_2_+Δ*C*_1_, Δ*C*_2_<*P*_2_+Δ*P*, *P*_3_<Δ*C*_3_, *E*_2_(0, 1, 0) is the ESS.
Scenario 2: When the conditions satisfy *P*_1_+*F*_1_<*S*_1_+*S*_2_+Δ*C*_1_, *P*_2_<Δ*C*_2_, Δ*C*_3_<*P*_3_+Δ*P*, *E*_3_(0, 0, 1) is the evolutionary equilibrium point of the system, and the ESS is {no regulation, illegal treatment, high-quality reproduction}.
Scenario 3: When the conditions satisfy *P*_2_+*F*_1_<Δ*C*_2_, *P*_2_+*F*_2_<Δ*C*_3_, *E*_4_(1, 0, 0) is the ESS.
Scenario 4: When the conditions satisfy *S*_1_+*S*_2_+Δ*C*_1_<*P*_1_+*F*_2_, Δ*C*_2_<*P*_2_+*F*_1_, *P*_2_+*F*_2_<Δ*C*_3_, *E*_6_(1, 1, 0) is the ESS.
Scenario 5: When the conditions satisfy *P*_1_<*S*_1_+*S*_2_+Δ*C*_1_, Δ*C*_2_<*P*_2_, Δ*C*_3_<*P*_3_, *E*_7_(0, 1, 1) is the evolutionary equilibrium point of the system, and the ESS is {nonregulation, legal treatment, high-quality reproduction}.

## 5. Simulation analysis

The aim of this paper is to improve the efficiency of construction waste recycling and build a greener construction waste recycling system. When construction units legally handle construction waste and recycling and reprocessing enterprises have high-quality reproduction, the construction waste recycling industry chain, which includes construction units and recycling and reprocessing enterprises, can develop smoothly. Considering the limited resources of government regulation, it is hoped that this study can reduce the cost of government regulation as much as possible while achieving the above goals.

Therefore, *E*_7_(0, 1, 1) is the optimal strategy combination after considering multiple factors. According to the above analysis, the evolutionary stability strategies and evolutionary paths vary across scenarios. To show the evolutionary trajectory and the final stable state more intuitively, a numerical simulation was carried out in MATLAB. The specific simulation process is divided into two parts: (1) Simulating the evolution results of the initial value and (2) Drawing up the second set of data on the basis of the initial values. The final evolution result of array two is the optimal ESS, which is *E*_7_(0, 1, 1) This section simulates the impact of changes in parameters such as policy subsidies, fines, and public favorability of the evolutionary results and evolutionary trajectories under this scenario.

### 5.1 Assignment of relevant parameters

Because there are many parameters involved in this study, the relationships among the parameters are complex. At the same time, in view of the lack of specific data on the recycling of construction waste at this stage, to reflect the real situation, this paper refers to relevant literature to determine some basic data [15, 43, 48]. This article also consulted the opinions of relevant industry experts and discussed and improved the collected data, as shown in S1 Table. Data that are difficult to quantify were also assigned, as shown in Table 5.

**Table 5:** Parameter assignment.

stakeholders	government						construction unit			Recycling and reprocessing enterprises		
parameters	Δ*C*_1_	S_1_	S_2_	F_1_	F_2_	P_1_	Δ*C*_2_	P_2_	ΔP	Δ*C*_3_	P_3_	ΔP
parameter assignment	0.06	0.04	0.04	0.09	0.09	0.03	0.03	0.04	0.01	0.05	0.04	0.01

**Fig 1:**
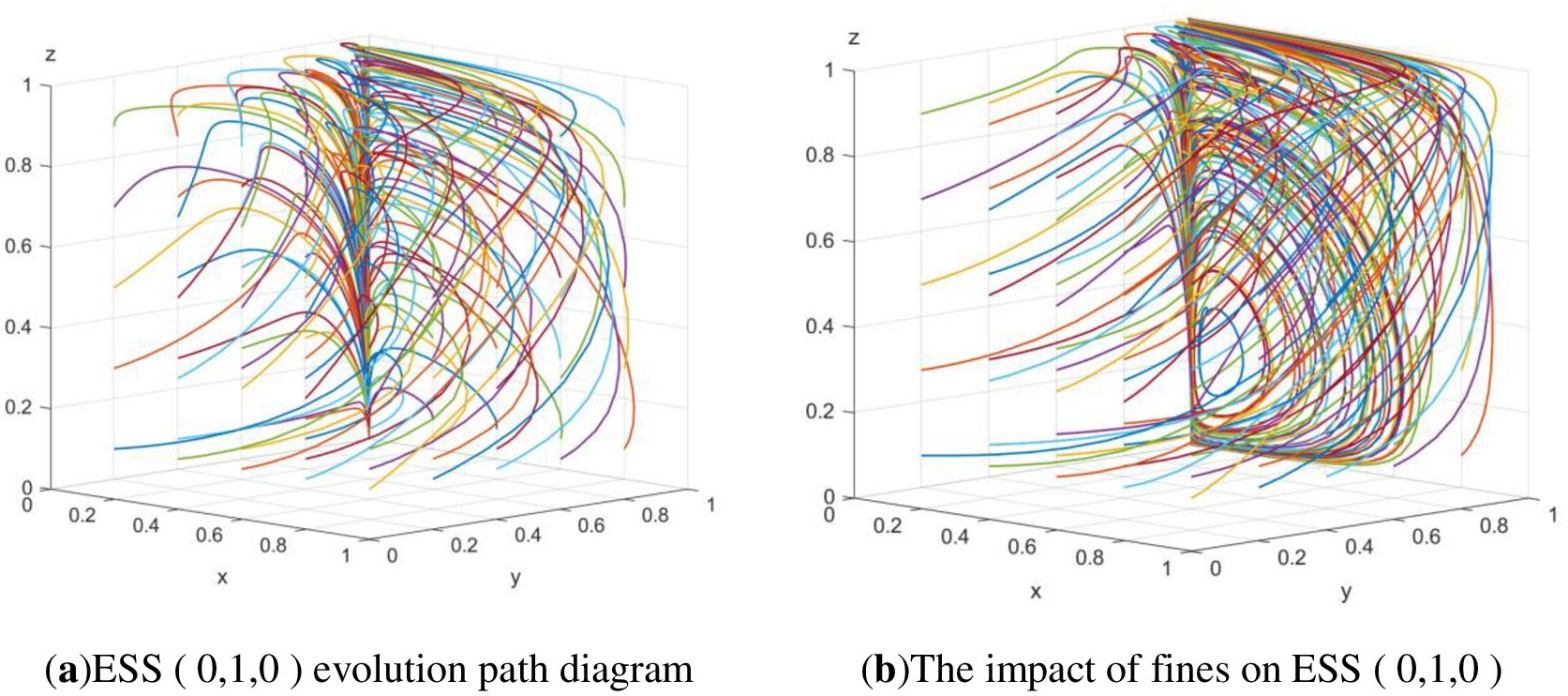
ESS (0, 1, 0) evolution path diagram and the impact of fines on the equilibrium point.

First, the initial data are formulated as an array: Δ*C*_1_ = 0.06, S_1_ = 0.04, S_2_ = 0.04, P_1_ = 0.03, Δ*C*_2_ = 0.03, P_2_ = 0.04, P = 0.01, Δ*C*_3_ = 0.05, P_3_ = 0.04, F_1_ = 0.09, and F_2_ = 0.09. The 100 strategy sets corresponding to the three stakeholders are randomly generated by MATLAB software to verify that the evolutionary equilibrium point under the initial data is *E*_2_(0, 1, 0), as shown in Fig 1(a). According to the above analysis, this point is not the optimal evolutionary stable point. At this time, the effect of low-carbon emission reduction is not obvious. This nonoptimal stable state urgently needs to be overcome. According to the simulation of Fig 1(b), increasing the amount of government fines F_1_ and F_2_ to 0.11 can obviously disrupt the nonoptimal stable state.

**Fig 2:**
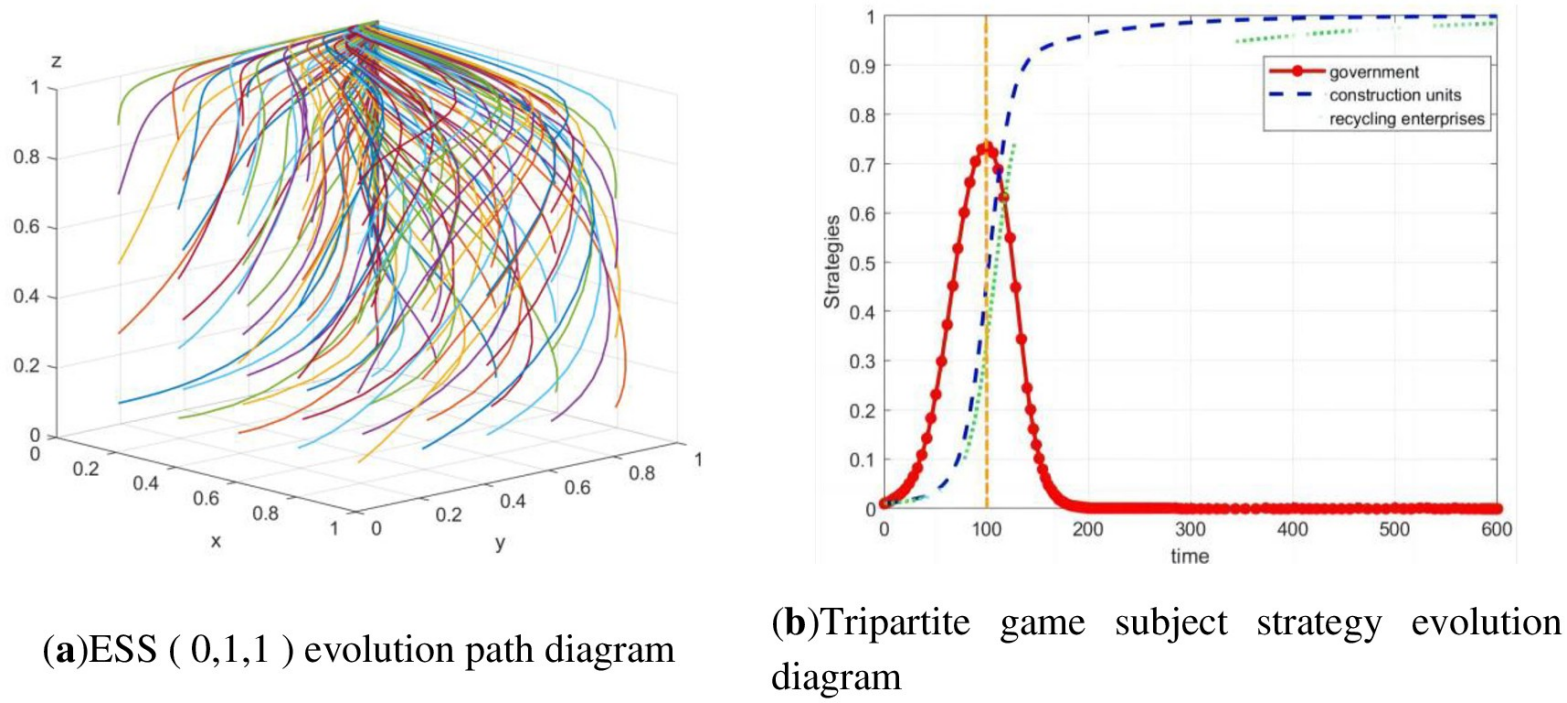
The evolution path diagram of ESS (0, 1, 1).

According to Scenario 5, array 2 was developed: Δ*C*_1_ = 0.06, S_1_ = 0.04, S_2_ = 0.04, *P*_1_ = 0.03, Δ*C*_2_ = 0.03, *P*_2_ = 0.04, *P* = 0.01, Δ*C*_3_ = 0.035, *P*_3_ = 0.04, *F*_1_ = 0.09, and *F*_2_ = 0.09. The 100 randomly generated strategy set from MATLAB verifies that array 2 satisfies the condition of E7 stability. In the case of array 2, the system is finally stable at *E*_7_(0, 1, 1), as shown in Fig 2(a).

In addition, the initial strategy of the three parties is set to (*x*, *y*, *z*) = (0.01, 0.01, 0.01) to observe the dynamic evolution process of the three-party strategy at this time. The practical significance of the initial strategy is that the government applies almost no regulation, the construction units almost do not legally treat their waste, and the recycling and reprocessing enterprises almost do not carry out high-quality reproduction. According to Fig 2(b), the government gradually evolved from almost no regulation (*x* = 0.01) to participation in regulation (*x* = 1), then returned to the state of nonregulation (*x* = 0), and finally remained stable. The strategy choices of construction units and recycling and reprocessing enterprises gradually evolve to *y* = 1 and *z* = 1 under the influence of government strategy fluctuations. Finally, the three-party evolutionary game system is stable at *E*_7_(0, 1, 1).

### 5.2 The impact of key parameters on stakeholder decision-making

The strategy of stakeholders involved in the model will change with the change in parameters, but the influence of each parameter on the strategy is unknown. Therefore, it is necessary to explore the specific impact of parameters on stakeholder behavior by controlling for certain variables. The parameters selected in this paper are divided into the following three levels:

Government level: Government fines *F*_1_, *F*_2_, government subsidies *S*_1_ and *S*_2_;
Enterprise level: The cost difference *ΔC*_2_ and *ΔC*_3_ of different strategies of the two parties;
Public level: Public participation increases the reputation benefits of game players *P*_1_, *P*_2_, *P*_3_, Δ*P*.

#### 5.2.1 The impact of F_1_ and F_2_ changes on stakeholder decision-making.

**Fig 3:**
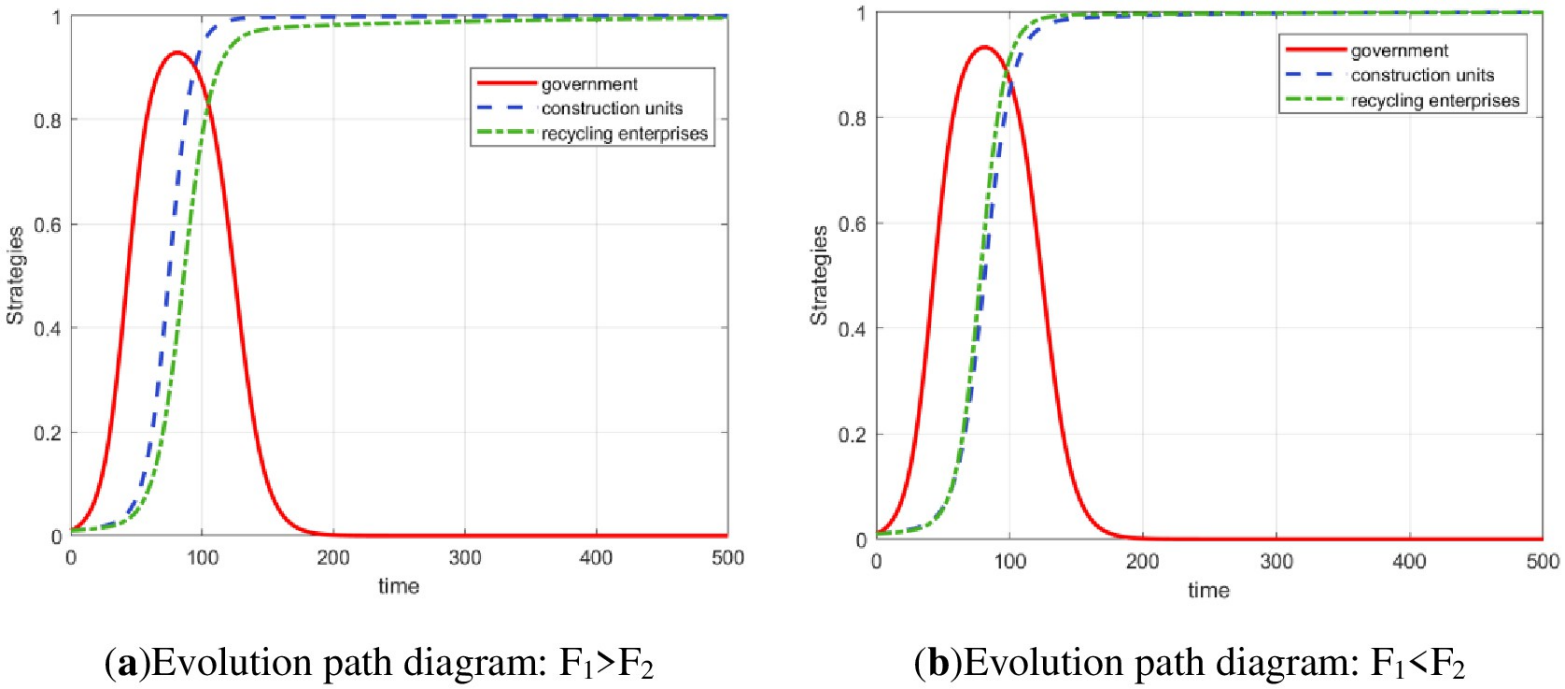
The influence of the change in penalties F_1_ and F_2_ on the game equilibrium.

Fig 3(a) and 3(b) are made by comparing the original data of Fig 2(b) with those of array 2. Fig 3(a) shows the evolutionary path of the stakeholder strategy when *F*_2_ is fixed and only 0.02 of *F*_1_ is added. When the penalty increases, the recovery unit evolves to a stable point faster. Due to the increase in fines, the pressure on government regulation costs has been alleviated to some extent, so the government will first evolve to the strategy of regulation, and the peak value of the government evolution curve will also increase significantly. In this case, even if the fines for recycling and reprocessing enterprises remain unchanged, the changes in the evolution path of the government caused by the fine *F*_1_ will also impact its behavior; i.e., the government raising one party’s fines will also have a deterrent effect on the other party, prompting the recycling and reprocessing enterprises to accelerate the speed of reaching a stable point.

Fig 3(b) is the evolution path map of stakeholders under the condition of only adding 0.02 of *F*_2_ and fixed *F*_1_ unchanged. It can be seen from the figure that when the penalty for the recycling and reprocessing enterprise increases, the speed of the enterprise’s evolution to the stable point is significantly accelerated, and it exceeds the evolution speed of the construction unit. The strategy evolution of government departments is the same as in Fig 3(a). Similarly, even if the penalty for the construction unit does not change, the speed of the construction unit approaching the stable point will increase.

#### 5.2.2 Impact of changes in S_1_ and S_2_ on stakeholder strategies.

**Fig 4:**
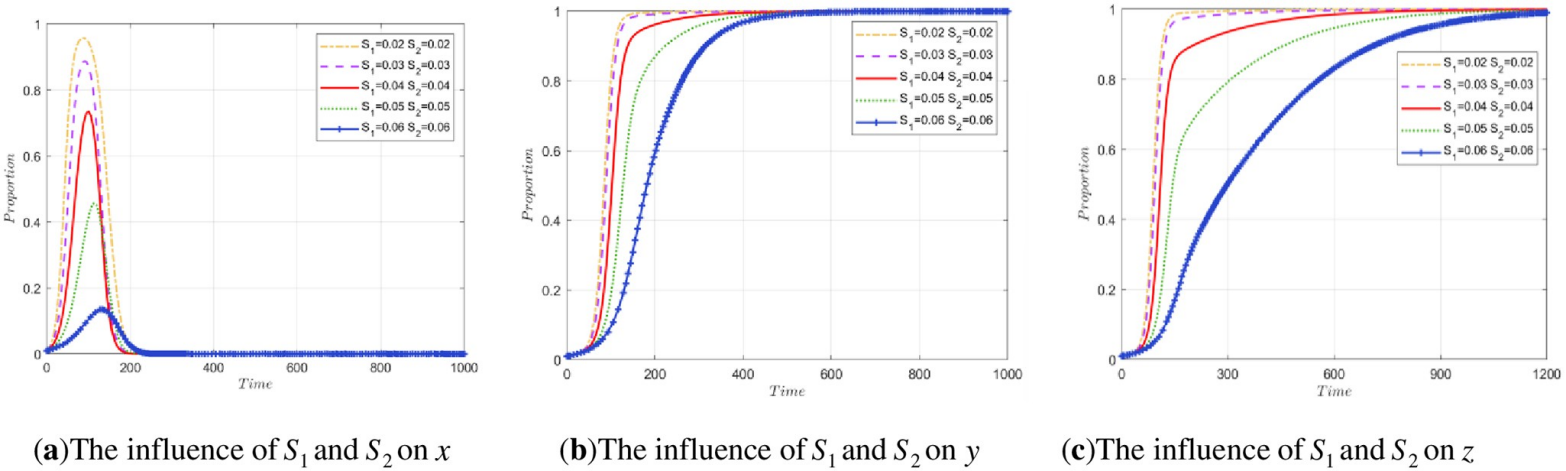
The impact of changes in S_1_ and S_2_ on stakeholder strategies.

Based on the parameter setting of array two, on this basis, it is assumed that the degree of government subsidies to the two enterprises is the same, so the parameters *S*_1_ and *S*_2_ are changed in the same direction to simulate the influence of *S*_1_ and *S*_2_ on the game balance. The evolution simulation of *S*_1_ and *S*_2_ changes on the game subject is shown in Fig 4.

Taking *S*_1_ = 0.04 and *S*_2_ = 0.04 as the contrast state, *S*_1_ and *S*_2_ increase by 0.01 and 0.02, respectively, and decrease by 0.01 and 0.02, respectively. The simulation results show that the smaller the subsidies given by the government to enterprises in the regulatory state is, the greater the peak value, which indicates that in the early stage of evolution, government departments tend to be regulated when the subsidies are low. In the later stage of evolution, as the two companies gradually approach the strategies of “legal treatment” and “high-quality reproduction, ” government departments will gradually tend to adopt “nonregulation” strategies to further reduce expenditures.

According to Fig 4(b) and 4(c), when the government’s subsidies increase, the inertia of the construction unit’s legal treatment and the recycling and reprocessing enterprise’s high-quality reproduction increase. At this time, the increase in subsidies will not increase their willingness to choose legal treatment and high-quality reproduction but will slow their evolution toward *y* = 1 and *z* = 1. The reason is that when the government regulates, the subsidies *S*_2_ and *S*_2_ increase, and even if both companies conduct ’illegal treatment’ and ’low-quality reproduction’, the loss of benefits caused by fines *F*_1_ and *F*_2_ will be reduced. At this time, the regulatory effect of fines on corporate behavior will be weakened, and some enterprises may take shortcuts instead of legal treatment and high-quality reproduction, which is not conducive to the strategic transformation of enterprises. Therefore, the appropriate reduction of subsidies by government departments in carrying out regulation is conducive to reaching ESS faster. However, subsidies should not be reduced without limits. Too low of a subsidy will put too much pressure on enterprises and cause negative effects.

#### 5.2.3 Impact of changes in S_1_ and S_2_ on stakeholder strategies.

To explore the impact of the cost difference Δ*C*_2_ of construction units and the cost difference Δ*C*_3_ of recycling and reprocessing enterprises on stakeholder strategies, this paper will conduct two sets of experiments.

**Fig 5:**
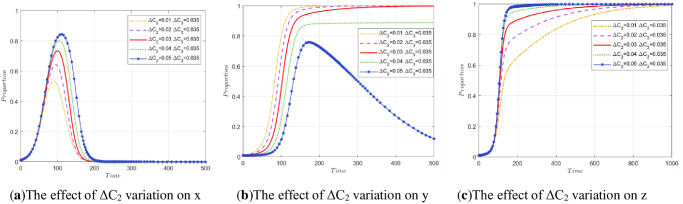
The impact of Δ*C*_2_ changes on stakeholder strategy.

The first group of experiments controls the value of Δ*C*_3_ unchanged and compares the strategy evolution obtained by array 2 to analyze the evolution of stakeholders when Δ*C*_2_ decreases by 0.01 or 0.02 and increases by 0.01 or 0.02. The simulation results of the three parties are shown in Fig 5(a)–5(c). A change in Δ*C*_2_ has little effect on government departments and recycling and reprocessing enterprises and affects the speed of their evolution to a stable point, as shown in Fig 5 (a) and 5(c). However, an increase in Δ*C*_2_ may lead to a decrease in the possibility of legal treatment of construction waste by construction units. When Δ*C*_2_>*P*_2_, the construction unit will ignore the government’s regulatory laws and regulations and evolve toward the strategy of illegal treatment.

**Fig 6:**
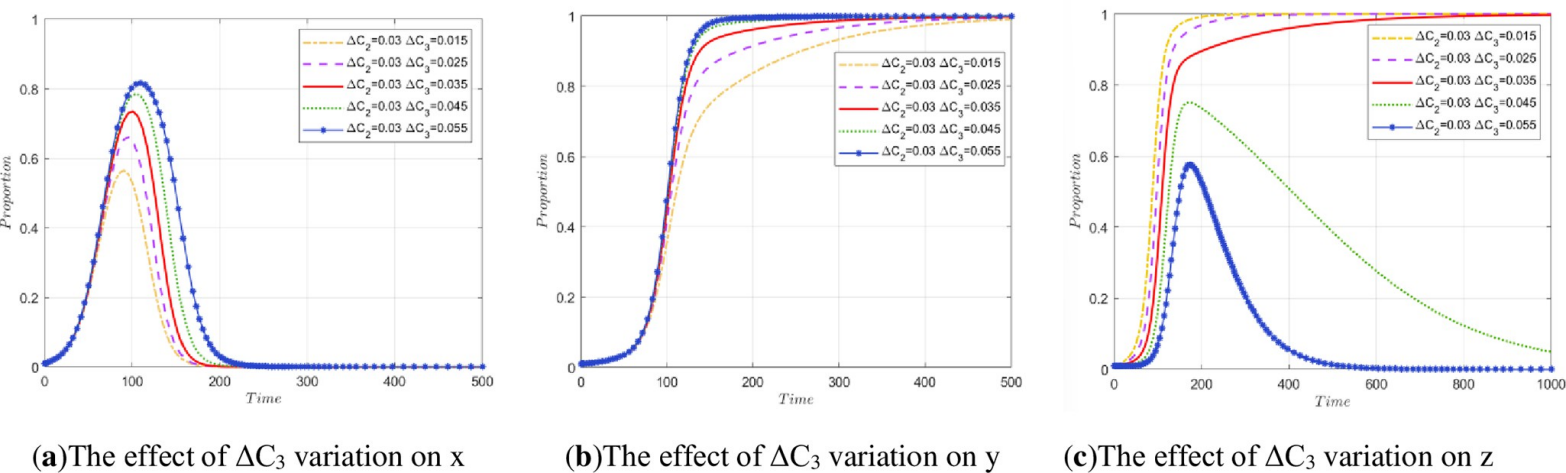
The impact of Δ*C*_3_ changes on stakeholder strategy.

The second group of experiments controls the value of Δ*C*_2_ unchanged and only changes the value of Δ*C*_3_. When Δ*C*_3_ = 0.035, a decrease of 0.01, a decrease of 0.02, an increase of 0.01, and an increase of 0.02 are still compared with the strategy evolution obtained by array 2. The impact of Δ*C*_3_ changes on stakeholder strategies is shown in Fig 6(a)–6(c). According to the evolution path map, the change in Δ*C*_3_ still only affects the speed of their evolution to a stable point, as shown in Fig 6(a) and 6(b). However, an increase in Δ*C*_3_ will affect the evolutionary results of recycling and reprocessing enterprises.

When the value exceeds the value of the P3, i.e., Δ*C*_3_>*P*_3_, the strategy of the recycling and reprocessing enterprise will not be stable with the high-quality reproduction strategy, and with the increase in Δ*C*_3_, the enterprise evolves to low-quality reproduction increasingly faster. When Δ*C*_3_<*P*_3_, the smaller Δ*C*_3_ is, the more willing the recycling and reprocessing enterprises are to choose the strategy of high-quality reproduction.

#### 5.2.4 Impact of P_1_, P_2_, and P_3_ changes on stakeholder strategies.

*P*_1_ represents the government’s gains in credibility and other aspects when the public participates in supervision. In addition to the degree of public participation, the influencing factors include the effectiveness of the government’s policies, the efficiency of government regulation, so the simulation experiment is carried out separately.

To explore the impact of changes in *P*_1_, *P*_2_, and *P*_3_ on stakeholder strategy, three simulation experiments are carried out. Simulation Experiment 1: Control *P*_1_ was unchanged so that the *P*_2_ and *P*_3_ of the two enterprises changed at the same time, and the impact on the evolutionary results of the three-party stakeholders was observed. Simulation Experiment 2: Control *P*_1_ is unchanged, the simulation of the evolution of array 2 is compared, the values of *P*_2_ and *P*_3_ are changed, and explore the evolution of the model when *P*_2_>*P*_3_ and the evolution of the model when *P*_2_<*P*_3_ In Simulation Experiment 3: Control, *P*_2_ and *P*_3_ remain unchanged and only *P*_1_ changes. The effect on the final stability is observed in this case.

(1) The impact of P_2_ and P_3_ co-movement on stakeholder strategy.

**Fig 7:**
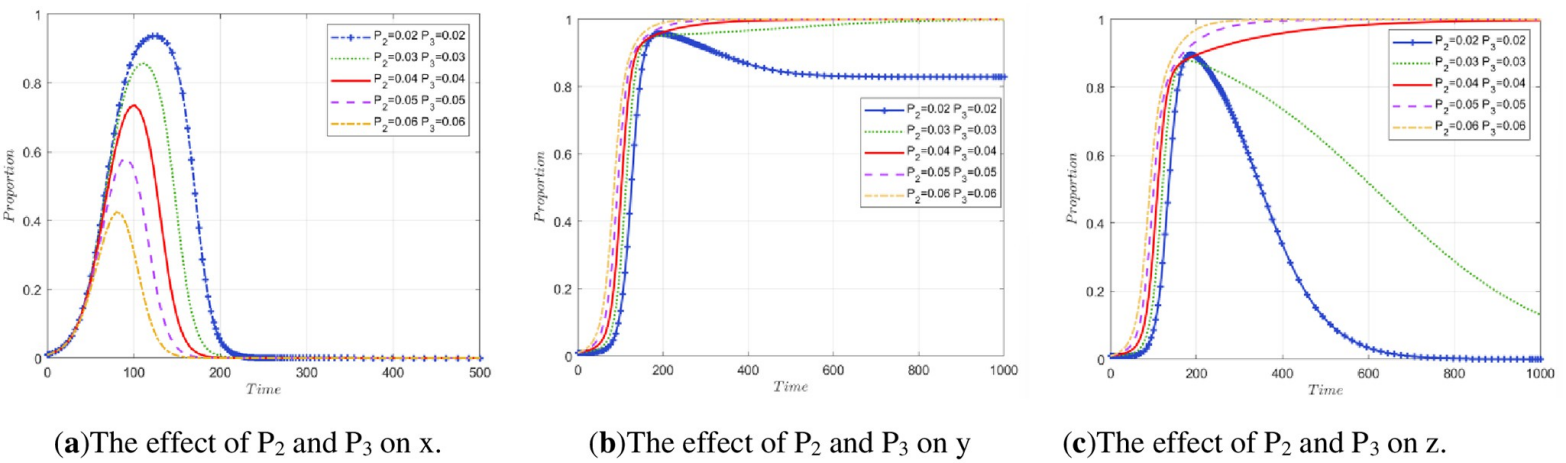
The impact of co-movement of P_2_ and P_3_ on stakeholder strategy.

A change in the degree of public participation in regulation will lead to an increase or decrease in *P*_2_ and *P*_3_ at the same time. The initial states are *P*_2_ = 0.04 and *P*_3_ = 0.04 in array 2, corresponding to increases or decreases of 0.01 and 0.02, respectively, at the same time. The simulation results are shown in Fig 7. From the diagram, it can be seen that when the degree of public participation decreases, the income of enterprises in terms of reputation, stock and so on decreases, the peak of the government evolution curve increases, and the time to reach the highest point increases. This reflects that when the degree of public participation in supervision is low, the government needs to assume the main regulatory responsibility. In the range of 100 to 150 cycles of evolution, the two enterprises rapidly increase, so the ratio of government regulation gradually decreases.

When public participation is too low, the two companies involved in this model will no longer approach *y* = 1, *z* = 1. When *P*_2_ = 0.03 and *P*_3_ = 0.03, the stable state of the model is broken, and with decreasing *P*_2_ and *P*_3_, the evolution result will no longer be *E*_7_(0, 1, 1) This reflects the importance of public participation supervision in regulating the behavior of enterprises. A lower degree of public participation reduces the enthusiasm of construction units for legal treatment and recycling and reprocessing enterprises for high-quality reproduction.

(2) The impact of different P_2_ and P_3_ on stakeholder strategy.

**Fig 8:**
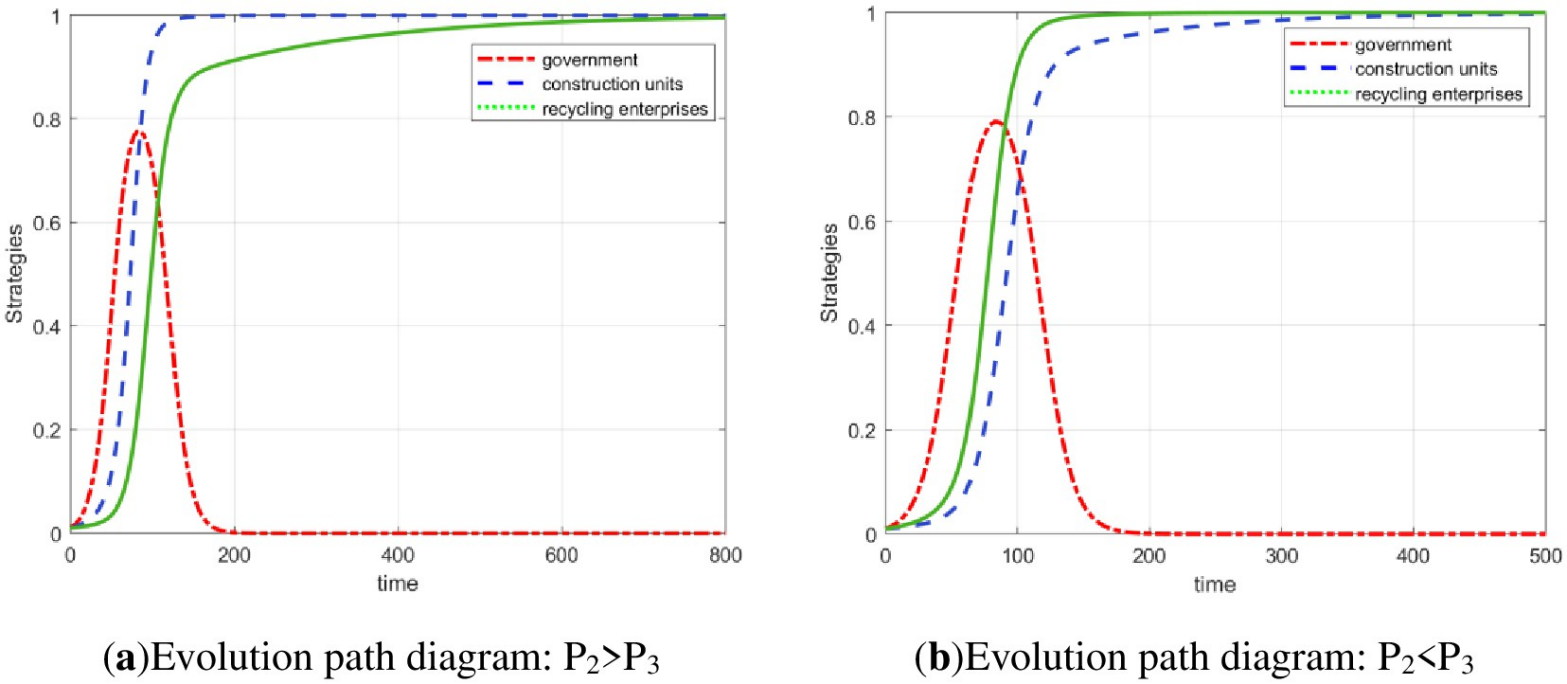
The influence of different temporal changes in P_2_ and P_3_ on the evolution process.

Fig 8(a) and 8(b) are made by comparing the original data of array 2 with Fig 2(b). Fig 8(a) shows the evolutionary path of the stakeholder strategy when *P*_3_ is fixed and only 0.02 of *P*_2_ are added (*P*_2_ = 0.06, *P*_3_ = 0.04). It can be seen from the figure that if *P*_2_>*P*_3_, the peak value of the government evolution curve increases slightly. The evolution speed of the construction unit accelerated, and it stabilized approximately 150 times. In contrast, an increase in *P*_2_ has a slight impact on recycling and reprocessing enterprises. When *P*_2_<*P*_3_, the evolution speed of recycling and reprocessing enterprises is also accelerated, and the time to reach stability is shorter. Specifically, the original performance stabilized after approximately 700 cycles, and after *P*_3_ increased, it stabilized after approximately 150 cycles. This once again proves that the greater the degree of public participation is, the more conducive it is for society to achieve ESS faster.

(3) The impact on stakeholder strategy only occurs when *P*_1_ changes.

**Fig 9:**
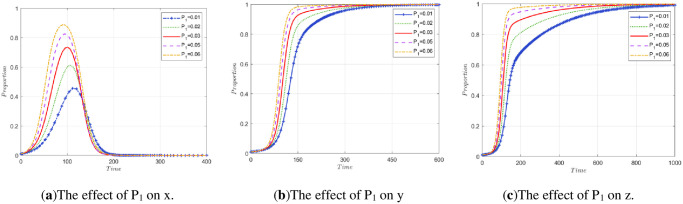
The impact of P_1_ change on stakeholder strategy.

Taking *P*_1_ = 0.03 in array 2 as the initial state while increasing by 0.01 and 0.02 and decreasing by 0.01 and 0.02, the simulation results are shown in Fig 9. It can be seen from the diagram that when *P*_1_ increases continuously, the government departments approach x = 1 at a faster rate in the early stage of evolution, and the peak value increases with increasing *P*_1_. After that, as construction units and recycling and reprocessing enterprises gradually tend to choose the strategies of legal treatment and high-quality reproduction, the government gradually returns to nonregulation based on considering lower fiscal expenditures, as shown in Fig 9(a). According to Fig 9(b), when *P*_1_ continues to increase, the speed at which construction units and recycling and reprocessing enterprises achieve stability is accelerated. The public should be encouraged to actively participate in supervision, taking into account the issue of obtaining low-carbon benefits as soon as possible.

#### 5.2.5 The impact of ΔP changes on stakeholder strategy

**Fig 10:**
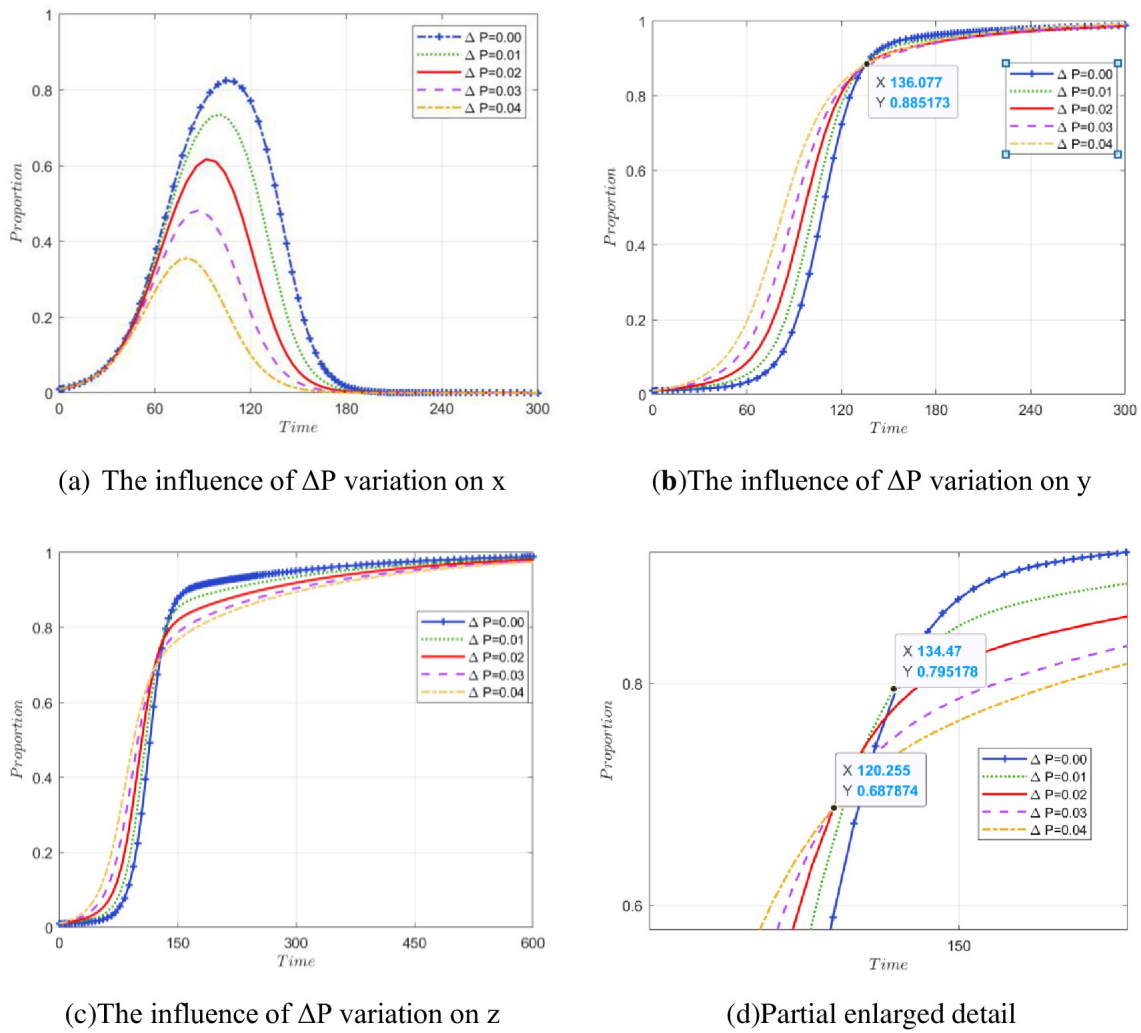
The impact of ΔP changes on stakeholder strategies.

ΔP indicates that without government regulation, when one party does not carry out green operations, the other party still insists on the additional added value of the gains in reputation, stock, and other aspects brought about by green operations. Fig 10(d) is a local enlargement of the boxed region in Fig 10(c). According to Fig 10(a), we can see that the evolution curve of government departments generally rises first and then falls. However, as ΔP increased from 0.00 to 0.04, the government’s rate of increase gradually accelerated and reached its peak faster. This means that when ΔP increases, the willingness of government departments to regulate decreases. When the degree of public participation is high, the government often chooses to reduce regulation appropriately. For the construction unit, the greater ΔP is, the faster the construction unit evolves to the stable point at the initial stage of evolution. When the government’s evolution curve reaches the peak, the government regulation ratio will decline, and the speed of the construction unit to the stable point will also decrease. When ΔP is large, a decrease in government regulation will lead to a decrease in the evolution speed of the construction unit to a stable point in the later stage of evolution, which is lower than that when ΔP is small; specifically, the number of evolutions in Fig 10(b) is approximately 136. Similarly, the impact of changes in ΔP on recycling and adding enterprises is also shown above, and the effect is more obvious. Comparing Fig 10(a) and 10(c), it can be seen that when ΔP = 0.04, compared with ΔP = 0.03, the government’s strategy evolution curve peaks earlier and then decreases. In Fig 10(c), in the vicinity of 120 times, the evolution curves of recycling and reprocessing enterprises in these two cases intersect. After the intersection, the curve at ΔP = 0.04 tends to be flatter and is surpassed by the curve represented by ΔP = 0.03. The reason is that when the regulatory ratio of government departments decreases earlier, the enthusiasm of recycling and reprocessing enterprises for high-quality reproduction will weaken, and the increase in inertia will lead to a decrease in the rate of evolution to a stable point.

### 5.3 Simulation results

Through the simulation analysis of the existing data, it is found that the process of recycling construction waste has a long way to go. Therefore, in the case of these data, adjusting the parameter F can effectively break the current stable state that is not conducive to the recycling of construction waste. On this basis, the optimal ESS is discussed; i.e., the optimal strategy is {nonregulation, legal treatment, high-quality reproduction}, and the model is optimized by adjusting the value of Δ*C*_3_ Based on the optimal strategy, the sensitivity of each relevant parameter is analyzed. The analysis results are as follows.

From the perspective of the government, the factors that affect the evolution speed and the final evolution results are fines and subsidies. On the one hand, an increase in fines can play a certain role in regulating corporate behavior and shorten the evolution process to a stable strategy; an increase in fines for one enterprise will also have an impact on the behavior of another enterprise. The strict treatment and recovery plan formulated by the government can establish common rules to effectively regulate the behavior of construction units and recycling and reprocessing enterprises and at the same time improve the confidence of both enterprises to maintain the stable development of the overall system of construction waste recycling. Therefore, government departments should impose high penalties on illegal construction units and recycling and reprocessing enterprises with low-quality reproduction to encourage enterprises that carry out green operations to reinforce their behavior [63]. On the other hand, low subsidies contribute to the development of the tripartite stability strategy. An increase in subsidies will increase the burden of fiscal expenditure, and the willingness of the government to regulate such expenditures will decrease. At this time, the two companies will probably be fluky, which is not conducive to the evolution to the optimal ESS. An increase in subsidies will offset the impact of fine losses on enterprises. At this time, the regulatory effect of fines on corporate behavior will be weakened. Some enterprises may take shortcuts and are unwilling to carry out legal treatment and high-quality reproduction, which is not conducive to their strategic transformation.

The government should actively introduce relevant policies on fines and subsidies, appropriately adjust the fines and subsidies, and promote the evolution rate to the optimal stable point in the case of avoiding fine-type income generation. The construction waste recycling process should be promoted to achieve carbon emission reduction and promote the sustainable development of the social environment.

From the perspective of participating enterprises, the cost difference between the two strategies seriously affects the final stable results. An increase in the cost difference between any two enterprises will slow the evolution speed of the enterprise to a stable point. After exceeding the threshold, the increase in the cost difference will lead to the enterprise refusing to reach the optimal ESS. However, the transportation, sorting, crushing, and reprocessing costs of construction waste are high, and in many areas, the cost of recycling and reprocessing products is often greater than that of raw building materials [64]. Therefore, while regulating the market, government departments can strive to promulgate subsidy policies for technological upgrading to alleviate the capital pressure on construction units and recycling and reprocessing enterprises.

From the perspective of the public, the parameters P_1_, P_2_, P_3_, and ΔP, which are affected by the degree of public participation, have a greater impact on the strategic choices of the three parties. The public pursues their own interests in social life. The public is exposed to social life as a direct beneficiary or victim of people’s environmental factors. It touches the vital interests of the public. At this time, the public will inevitably choose to actively supervise, and the degree of participation will increase significantly. The increase in reputation brought about by the public will have a certain incentive effect on the behavior of the two parties’ enterprises and the government. To a certain extent, public participation will promote the evolution of the construction waste recycling process to the optimal stable state. It can be seen that the public’s basic cognition, value, and importance cognition, and participation input have a positive direct effect on environmental protection. Therefore, the government should first consider raising public awareness and enthusiasm to participate and then further promote the transformation of value and importance cognition to participation behavior intention to increase the public’s participation in management behavior intention.

Considering public participation, the choice of government strategy shows an inverted U-shaped change, which is different from the expected results and other scholars’ research. The peak value of the U-shaped curve is affected by the degree of public participation. A higher degree of public participation can increase government regulatory pressure, and the government’s willingness to regulate will reduce the peak of the U-shaped curve.

## 6. Discussion

In the literature, few studies have considered the importance of government participation, waste-related processing enterprises, and the importance of public participation factors [10, 11, 20, 21, 43]. Through market research and a literature review, the high-quality reproduction of recycling and reprocessing enterprises plays a very important role in the recycling process of construction waste. However, in the existing research on the management of stakeholders in the recycling of construction waste, few studies have focused on the production quality of recycling and reprocessing enterprises.

According to the research gaps mentioned above, this paper constructs a tripartite evolutionary game model composed of government, construction units, and recycling and reprocessing enterprises under the influence of public participation factors. The strategy selection of production quality in the reproduction process of recycling and reprocessing enterprises is studied. We find the following results.

Government fines and subsidies affect the speed of evolution and the final evolution results. First, an increase in fines can shorten the evolution process to a stable strategy and play a certain role in regulating the behavior of enterprises. The fine imposed on one party’s enterprise also has an impact on the other’s enterprise. Second, we find that lower subsidies contribute to the development of the tripartite stability strategy. However, in the existing evolutionary game model analysis of government participation in energy conservation and emission reduction, some scholars believe that an increase in the government subsidy coefficient is conducive to improving the speed of evolution of other subjects to the optimal stable point. The greater the possibility of choosing collaborative emission reduction is, the faster the convergence speed of the system [65]. They believe that the possibility of enterprises on both sides choosing an active participation strategy is positively correlated with the cost subsidy coefficient [66]. Increasing subsidies is conducive to construction waste recycling enterprises choosing green operation behavior [67]. Some scholars also believe that government subsidies have little impact on the decision-making behavior of construction waste recycling enterprises [68]. This finding is different from the results of this study.

At the enterprise level, the final stable result is strongly affected by the cost difference between the two strategies of the enterprise. The increase in the cost difference between the two strategies of the enterprise will slow the evolution of the enterprise to a stable point. After the increase in the cost difference exceeds the threshold, the enterprise will refuse to evolve to the optimal ESS, which is reflected in both enterprises. At present, other studies in this field have shown such results [15], and some scholars believe that future research should focus on intelligent demolition methods for buildings and optimization of cost‒benefit processes [69], which further verifies the rationality of this study.

P1, P2, P3, and ΔP, which reflect the degree of public participation, have a great influence on the strategic choices of the three parties. The increase in reputation caused by public participation will have a certain incentive effect on the strategic choices of the three parties in the game model. Most of the existing research focuses on the view that when the benefits of public participation in construction waste disposal are greater than the losses of nonparticipation, the public will increase the willingness to participate [44], and public participation can give the government and enterprises a certain deterrent [52], thus promoting the smooth and efficient process of construction waste recycling [70]. Few articles describe the detailed evolution process of public participation in the strategy selection of the three parties of the game theme set in this paper. An inverted U-shaped curve of government behavior under public participation is found in this paper, and the peak value of the curve is affected by the degree of public participation. This finding is different from the expected results and the content of other scholars’ research.

The research of this paper is based on the assumption of relative idealism. There are only three parties involved in the construction waste treatment process. Although the game is carried out under the supervision of the fourth-party public, there are still some stakeholders in the construction waste disposal stage. It is not considered by environmental protection organizations, carbon emission monitoring agencies, etc. [71–73]. In addition to the data obtained from the literature, this paper has limitations because of the use of data collected by relevant experts for simulation.

In future research, we will continue to improve the model in view of the shortcomings of these two aspects. Combined with more cases, more other influencing factors will be included in the game model so that the model can more clearly reflect the construction waste treatment problem against the background of low carbon in reality.

The related research on digital technology in today’s society is a hot topic [74, 75], and future research on construction waste recycling can be combined with digital technology. Using digital technology to build a construction waste supervision system can improve construction waste recycling [76, 77]; applying digital technology to construction waste generation, transportation, processing, and other aspects of the data collection process and statistical analysis to optimize resource management strategies and predict future trends [78]; using AI image recognition and other technologies, intelligent classification and sorting of construction waste are carried out to improve the accuracy and efficiency of classification [79]; using digital technology to carry out education and training activities on the utilization of construction waste resources and improve the awareness and skills of the public and relevant practitioners on the utilization of resources [80]; and encouraging digital technology enterprises to carry out cross-border cooperation with traditional construction waste treatment enterprises to jointly develop new technologies, new products and new services. The combination of construction waste and digital technology can not only improve the efficiency and quality of construction waste resource utilization but also promote the development of a circular economy and the construction of sustainable cities. In the next step, we hope to combine digital technology with the resource utilization process for more in-depth research.

## 7. Conclusions and management recommendations

This paper constructs an evolutionary game model of construction waste treatment composed of a government, construction units, and recycling and reprocessing enterprises and analyzes it with respect to public participation. Through the simulation analysis of array data, it was found that the current construction waste treatment industry has not yet reached the most stable strategy suitable for the sustainable development of society. Therefore, in the case of array one, the parameters are adjusted, the optimal construction waste treatment strategy group is discussed, and the sensitivity of each relevant parameter is analyzed on the basis of the optimal strategy. In this paper, the numerical simulation evolution process is used to explore the influence of several key parameters on the decision-making of each stakeholder considering the public participation factor. The conclusions and recommendations of this paper are as follows.

### 7.1 Conclusion

Through the simulation analysis of the existing data, it is found that it is difficult for the existing data to achieve the optimal stable state, and there is ample room for improving construction waste recycling processes. This finding shows that China’s construction waste recycling is still in its infancy. By adjusting the parameters, the optimal strategy is {nonregulation, legal treatment, high-quality reproduction}. Based on the optimal strategy, the sensitivity of each relevant parameter is analyzed.

1. An increase in fines can play a certain role in regulating corporate behavior and can shorten the evolution process to a stable strategy [63].
2. A lower subsidy cost can promote the evolution of the strategy selection of the three parties to the optimal stable point. This finding is different from those of some existing studies [65–68]. We believe that an increase in subsidies will increase the burden of fiscal expenditures, and the government’s willingness to supervise will decrease. An increase in subsidies will offset the impact of penalty loss on enterprises, and the regulatory effect of penalties on enterprise behavior will be weakened. After analysis, we found that the results of this study are more in line with the actual situation and can provide reference suggestions for the government’s next policy formulation.
3. The cost difference between the two strategies seriously affects the construction waste recycling process [3, 15, 69]. An increase in the cost difference between any two enterprises will weaken the willingness of the enterprise to carry out green operations. After the cost difference exceeds the threshold, the enterprise will refuse to carry out green operations.
4. The degree of public participation has a greater impact on the strategic choices of the three parties [51]. An increase in reputation brought about by the public will have a certain incentive effect on corporate and government behavior [52].
5. When considering public participation, the peak value of the inverted U-shaped curve of government strategy choice is affected by the degree of public participation. Under the contrast effect, an increase in public participation will reduce the willingness for government supervision. The two contain each other, and a higher degree of public participation can reduce some of the government’s regulatory pressure, which can have a significant impact on the choice of corporate behavior strategies.

### 7.2 Management recommendations

To promote the collaboration of various stakeholders in the utilization of construction waste resources and improve the utilization of construction waste resources, this paper proposes the following suggestions:

1. As the publisher of policies and regulators of society, to establish a low-carbon sustainable development society and a good public image, the government should introduce corresponding policies on construction waste treatment and appropriately adjust the fines and subsidies [81]. The fines can be appropriately increased but not excessively increased to avoid fine-type income generation. The government can also encourage enterprises in construction waste recycling to use innovative emerging technology and equipment and talent [3] to reduce the green operation cost of enterprises. In addition, the government should also consider raising public awareness and enthusiasm for participation to encourage the public to actively participate in supervision [82] and to promote the transformation of value and importance cognition to participation behavior intention to enhance the public’s participation in management behavior intention [16].
2. As the main body of construction waste disposal, the two parties should take responsibility. Construction units should opt for the legal treatment of construction waste, and recycling and reprocessing enterprises should also carry out high-quality reproduction. Enterprises can reduce the cost of legal treatment by means of technological innovation and promote the evolution of their strategic choices in the direction that is most conducive to the development of a low-carbon society. At the same time, it can also enhance the reputation and recognition of enterprises and improve their core competitiveness [43].
3. As direct victims of environmental pollution, the public has been in a passive position in construction waste disposal, and their sense of participation is not strong [16]. The government should actively carry out low-carbon environmental protection campaigns, stimulate the public’s sense of ownership, encourage the public to actively participate in supervision, and increase public awareness to ensure a good social governance atmosphere [43]. The role of various social public entities in the treatment of construction waste, such as establishing a new media information disclosure mechanism to expose corporate violations and industry associations identifying violations of the nongreen behavior of enterprises, should be fully considered [52].

### 7.3 Social implications

1. Resource savings:
  Through resource utilization, construction waste can be converted into new building materials or products, thereby reducing the exploitation and consumption of natural resources [23, 31]. Therefore, through the research of this paper, we can explore the key factors for improving the utilization efficiency of construction waste to achieve further resource conservation.
2. To promote the development of a circular economy:
  Cooperation between stakeholders can promote the development of the construction waste resource utilization industry [47] and promote the development of a circular economy [1, 3, 70]. Through model construction and simulation, the key factors affecting the recycling effect of construction waste can be identified, and corresponding rectification suggestions can be proposed to help China’s construction industry reduce the overexploitation and consumption of natural resources and realize the sustainable utilization of resources [9, 83].
3. Enhancing environmental quality
  The resource utilization of construction waste can reduce the pollution and damage of construction waste to the environment and improve environmental quality [41, 43]. Cooperation between stakeholders can promote the establishment of a more effective construction waste treatment system [38, 39] and improve treatment efficiency and quality to further improve environmental quality.
4. Enhancing the sense of social responsibility:
  By analyzing the role, responsibility and synergy of stakeholders in the process of construction waste resource utilization [15, 46], the awareness of social responsibility of all parties can be enhanced. This helps to form a social atmosphere and motivation to jointly promote the utilization of construction waste resources.
5. Promoting industrial upgrading and technological innovation:
  The research in this paper can enhance the upgrading of the construction waste resource utilization industry and the driving force of technological innovation. Under technological innovation, emerging technologies can not only reduce the cost of high-quality reproduction processes for recycling and reprocessing enterprises but also improve the quality of reproduced products, which is conducive to the healthy development of waste treatment systems in China’s construction industry [79, 84]. We suggest that the government can also support the technological innovation and industrial upgrading of enterprises through policy support and capital investment and promote the rapid development of the construction waste resource utilization industry.

### 7.4 Limitations and shortcomings

At present, there are still some limitations in this study.

This study is based on relatively ideal assumptions and involves only three parties involved in the construction waste treatment process. Although this paper is a game under the supervision of the public, which is the fourth party, there are still some stakeholders in the waste disposal stage of the construction that are not taken into account, such as environmental protection organizations and carbon emission monitoring institutions. Therefore, establishing a more realistic model that considers the interests of all parties involved in the main body will be one of the next research priorities.

In addition to the data obtained from the literature, this paper has limitations because of the use of data collected by relevant experts for simulation. To better match reality, more data collection is needed, and the next research direction should be moving closer toward empirical analysis.

In future research, we will continue to improve the model in view of the mentioned shortcomings. With more cases, more other influencing factors will be included in the game model so that the model can more clearly and realistically reflect the construction waste treatment problem against the background of low carbon.

## Supporting information

S1 Fig Collaborative mechanism of construction waste recycling. (TIF)

S2 Fig Considering the interest demands and game relationship of the three parties under the factor of public participation. (TIF)

S1 Table Statistics table of parameter values. (DOCX)
