# Supplementary figures and images for: Evolutionary game and simulation analysis of construction waste recycling from the perspective of stakeholders
Source: PLoS One. 2024 Aug 27;19(8):e0307652. doi: 10.1371/journal.pone.0307652 (PMC11349105; doi:10.1371/journal.pone.0307652)

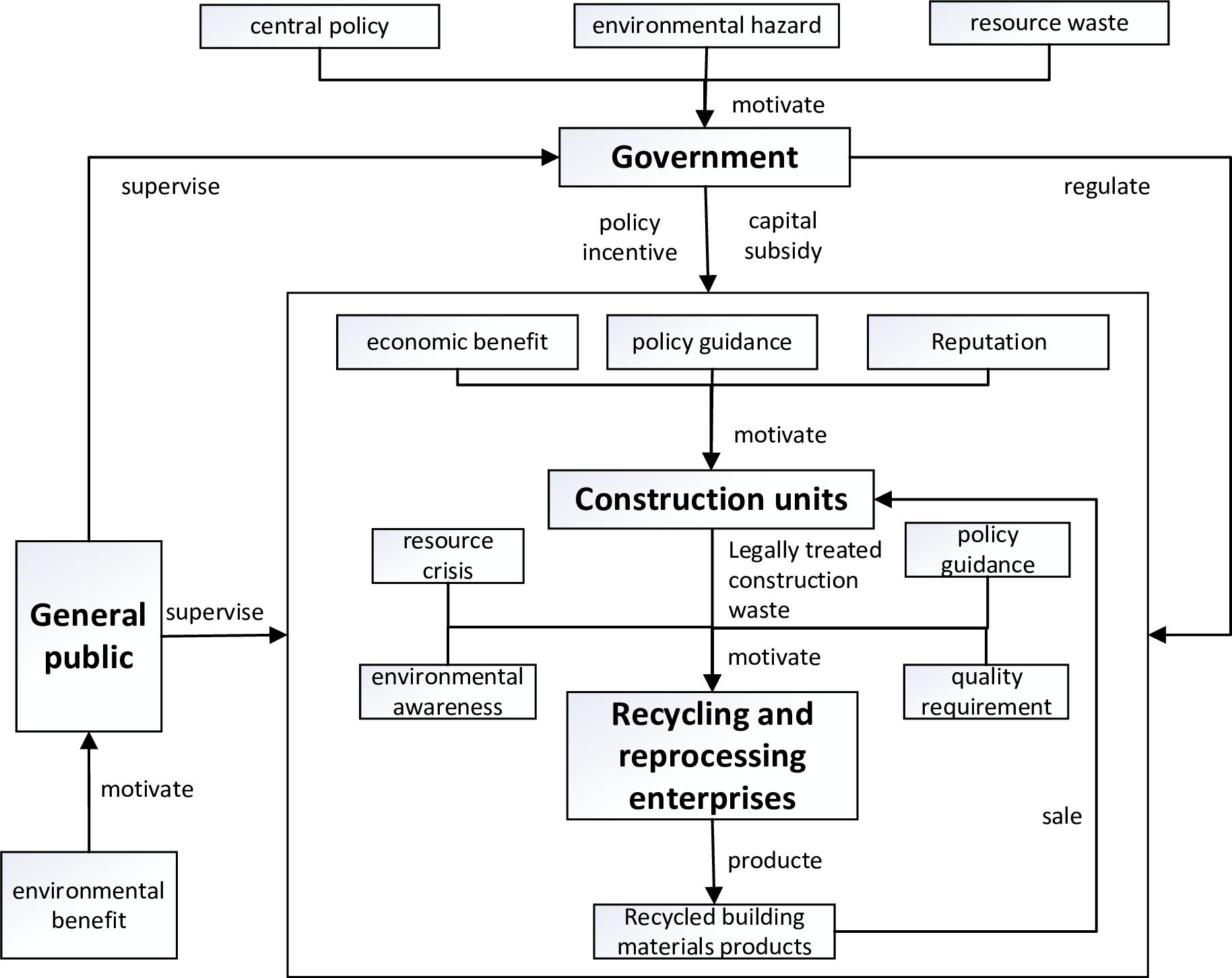

Supplement: S1 Fig — (TIF) [file pone.0307652.s001.tif]

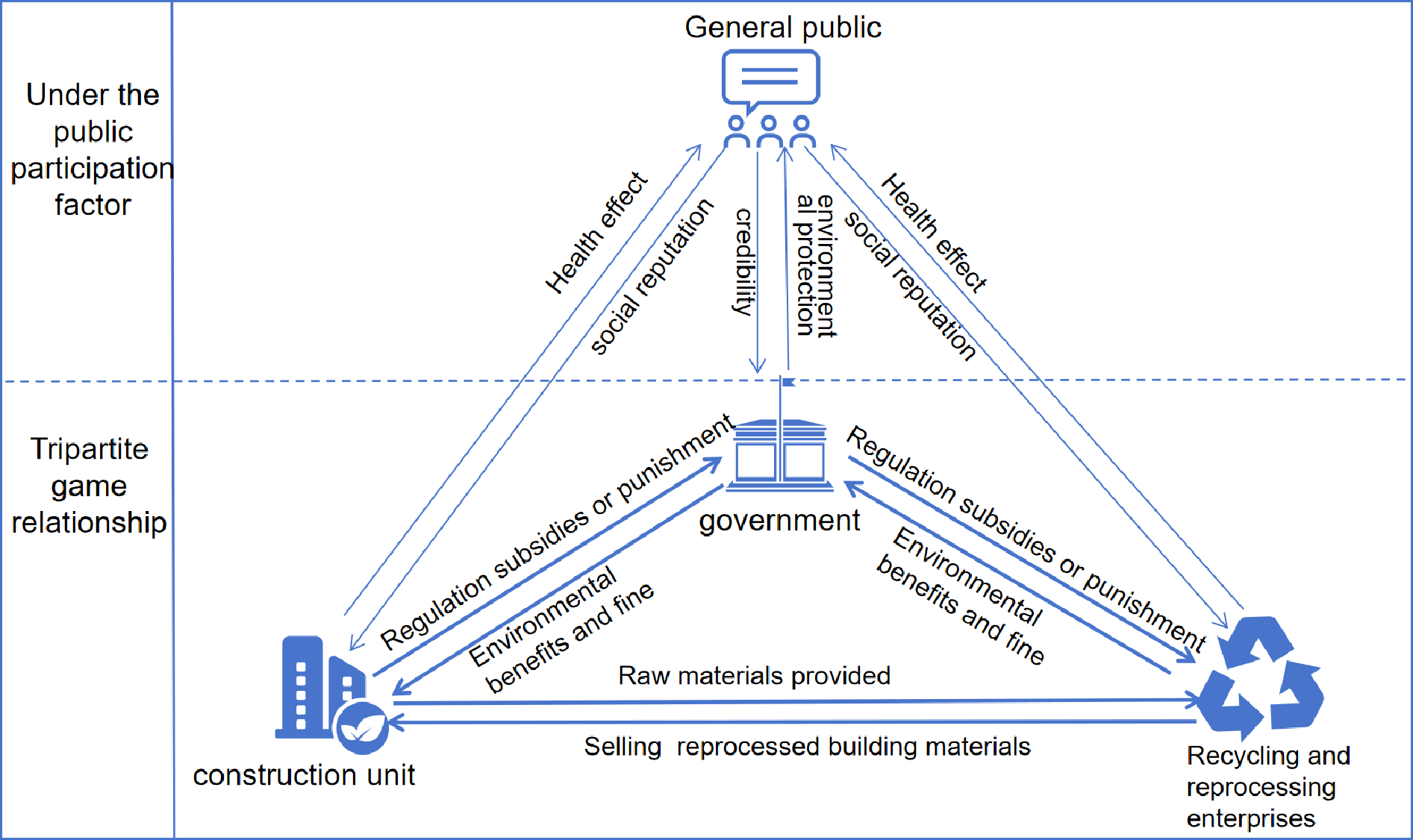

Supplement: S2 Fig — (TIF) [file pone.0307652.s002.tif]
