# Supplementary material for: Evolutionary game and simulation analysis of construction waste recycling from the perspective of stakeholders
Source: PLoS One. 2024 Aug 27;19(8):e0307652. doi: 10.1371/journal.pone.0307652 (PMC11349105; doi:10.1371/journal.pone.0307652)
Supplement: S1 Table — (DOCX) [file pone.0307652.s003.docx]

Data S_2_ and$\Delta C_{3}$ obtained according to the literature [15], data $\Delta C_{1}$and F_2_ are obtained after reviewing the literature [43,44] and adjusting the data after discussion by the research team. The remaining data according to the above four data, the questionnaire was distributed to the relevant researchers to collect scores.

|  | **Government** | | | | | | **construction unit** | | **Recycling and reprocessing enterprises** | |  |
| --- | --- | --- | --- | --- | --- | --- | --- | --- | --- | --- | --- |
|  | $\Delta C_{1}$ | S_1_ | S_2_ | F_1_ | F_2_ | P_1_ | $\Delta C_{2}$ | P_2_ | $\Delta C_{3}$ | P_3_ | ΔP |
| Data 1 | 0.06 | 0.1 | 0.04 | 0.09 | 0.09 | 0.03 | 0.04 | 0.04 | 0.05 | 0.04 | 0.01 |
| Data 2 | 0.06 | 0.04 | 0.04 | 0.09 | 0.09 | 0.05 | 0.03 | 0.05 | 0.05 | 0.03 | 0.02 |
| Data 3 | 0.06 | 0.04 | 0.04 | 0.08 | 0.09 | 0.01 | 0.03 | 0.03 | 0.05 | 0.03 | 0.005 |
| Data 4 | 0.06 | 0.03 | 0.04 | 0.10 | 0.09 | 0.04 | 0.02 | 0.04 | 0.05 | 0.04 | 0.01 |
| Data 5 | 0.06 | 0.05 | 0.04 | 0.09 | 0.09 | 0.02 | 0.05 | 0.03 | 0.05 | 0.04 | 0.005 |
| Data 6 | 0.06 | 0.04 | 0.04 | 0.09 | 0.09 | 0.03 | 0.04 | 0.05 | 0.05 | 0.05 | 0.007 |
| Data 7 | 0.06 | 0.04 | 0.04 | 0.08 | 0.09 | 0.02 | 0.05 | 0.04 | 0.05 | 0.02 | 0.01 |
| Data 8 | 0.06 | 0.05 | 0.04 | 0.09 | 0.09 | 0.05 | 0.04 | 0.04 | 0.05 | 0.02 | 0.02 |
| Data 9 | 0.06 | 0.06 | 0.04 | 0.10 | 0.09 | 0.06 | 0.04 | 0.03 | 0.05 | 0.04 | 0.01 |
| Data 10 | 0.06 | 0.03 | 0.04 | 0.09 | 0.09 | 0.03 | 0.04 | 0.02 | 0.05 | 0.05 | 0.005 |
| Average data | 0.06 | 0.043 | 0.04 | 0.09 | 0.09 | 0.034 | 0.038 | 0.037 | 0.05 | 0.036 | 0.0102 |
| Approximate value | 0.06 | 0.04 | 0.04 | 0.09 | 0.09 | 0.03 | 0.04 | 0.04 | 0.05 | 0.04 | 0.01 |

**S 3 Table. Statistics table of parameter values**
